# GALNT12 suppresses the bone-specific prostate cancer metastasis by activating BMP pathway via the O-glycosylation of BMPR1A

**DOI:** 10.7150/ijbs.91925

**Published:** 2024-01-27

**Authors:** Yang Yang, Meng Ding, Haoli Yin, Wei Chen, Hongwei Shen, Wenli Diao, Lin Yang, Haixiang Qin, Weidong Gan, Xuefeng Qiu, Hongqian Guo

**Affiliations:** 1 Department of Urology, Drum Tower Hospital, Medical School of Nanjing University, Institute of Urology, Nanjing University, China.; 2 Department of Urology, The Third Affiliated Hospital of Kunming Medical University, Tumor Hospital of Yunnan Province, Kunming, China.

**Keywords:** prostate cancer, bone metastasis, O-glycosylation, GALNT12, BMPR1A

## Abstract

Bone metastasis caused the majority death of prostate cancer (PCa) but the mechanism remains poorly understood. In this present study, we show that polypeptide N-acetylgalactosaminyltransferase 12 (GALNT12) suppresses bone-specific metastasis of PCa. GALNT12 suppresses proliferation, migration, invasion and cell division ability of PCa cells by activating the BMP pathway. Mechanistic investigations showed that GALNT12 augments the O-glycosylation of BMPR1A then actives the BMP pathway. Activated BMP signaling inhibits the expression of integrin αVβ3 to reduce the bone-specific seeding of PCa cells. Furthermore, activated BMP signaling remolds the immune microenvironment by suppressing the STAT3 pathway. Our results of this study illustrate the role and mechanism of GALNT12 in the process of bone metastasis of PCa and identify GALNT12 as a potential therapeutic target for metastatic PCa.

## Introduction

Prostate cancer (PCa) ranks as the second most prevalent cancer type among men worldwide. While localized PCa presents a 5-year survival rate of 100%, approximately one-third of PCa patients progress to advanced metastatic PCa, which carries a significantly worse 5-year survival rate of less than 30% 1, 2. Cancer cells frequently exhibit the proclivity to metastasize to specific organs. For example, sarcoma often metastasizes to lung, colorectal cancer to liver and lung, and bone is the most commonly metastatic site of PCa 3. Notably, the majority of advanced PCa patients exhibit multiple metastases, with more than 80% developing bone metastases. Liver (10%), lung (7%), and brain (3%) metastases follow in prevalence 4-6. Visceral metastasis, such as liver, lung, and brain metastases, are predominantly detected in PCa patients with neuroendocrine differentiation or castration-resistant PCa^6^. The process of PCa cells colonizing bone is intricate and multifaceted, involving dissociation from the primary tumor, dissemination through the circulation, seeding in distant metastatic organs, and subsequent restoration of proliferation to form metastatic lesions 2. However, the molecular mechanisms underlying the heightened propensity of PCa cells to metastasize to bone remain poorly understood.

As a common post-translational modification, O-glycosylation is involved in a variety of biological process such as intercellular interaction 7, 8, protein stability 9, 10, signal transduction 11 and protein activity 12, 13. Mucin-type O-glycosylation is the most common O-glycosylation and it is initiated by the polypeptide N-acetylgalactosaminyl transferases (GALNTs) which transfers GalNAc from UDP-GalNAc to the serine (Ser)/threonine (Thr) residues to form GalNAcα1-O-Ser/Thr linkage in O-glycoproteins 14. Aberrant O-glycosylation and GALNTs has been proved to be associated with tumor development and metastasis in different cancers. For instance, GALNT8 could suppress the metastasis of breast cancer via suppressing the EGFR signaling pathway 15. GALNT4 promoted the O-GalNAc modification of TGF-β type Ⅰ and Ⅱ receptor in breast cancer and then suppressed the dimerization and activity of TGF-β receptor restraining the migration and invasion of cancer cells 16. GALNT2 promoted the invasion of colorectal cancer cells via mediating the O-GalNAcylation of AXL 17. To date, 20 GALNT family members in human have been identified, but the expression levels and substrate specificity of these GALNTs are often divergent under different cellular context, adding the complexity to explore the functional roles and mechanisms of GALNTs.

In the present study, we explored the mechanism of bone-specific metastasis of PCa, and proved that GALNT12 suppressed bone-specific metastasis of PCa. Specifically, GALNT12 promoted the O-GalNAcylation of BMPR1A and subsequently activated BMP signaling, ultimately suppressed bone-specific metastasis of PCa cells in three ways. On the one hand, GALNT12-BMP signaling directly inhibited the proliferation, migration, and invasion abilities of PCa cells. On another hand, GALNT12-BMP signaling decreased the expression of integrin αVβ3 to impede the adhesion and spread of PCa cells to bone matrix. On the third hand, GALNT12-BMP signaling could modulate the immune microenvironment of bone via suppressing STAT3 signaling to prevent the survival of PCa cells in bone.

## Materials and Methods

### Clinical tissue samples

12 PCa biopsy samples, 12 bone metastasis lesions, 2 lung metastasis lesions and 3 brain metastasis lesions samples were obtained from metastatic PCa patients undergoing biopsies or surgical resection from 2011 to 2021. This study was consistent with the Declaration of Helsinki principles and approved by the Ethics Committee of Drum Tower Hospital, Medical School of Nanjing University.

### Generation of GALNT12 catalytic mutant and BMPR1A O-GalNAcylation mutant

Glycosyltransferase 1 (GT1) motif which conserved among all 20 human GALNT family members 18. Three amino acid residues in GT1 motif were substituted to produce the GALNT12 catalytic mutant, including Asp228→Asn, His230→Asp, and Glu232→Gln.

The O-GalNAcylation sites of BMPR1A by GALNT12 were predicted by ISOGlyP (https://isoglyp.utep.edu/) 19. Two mutants were generated by substituting Thr49→Ala and Thr137→Ala, respectively.

### Animal studies and isolation of lung and bone-derived cells

8-week-old male C57/B6 mice were purchased from GemPharmatech Company (Nanjing, China). For the isolation of lung and bone-derived cells, 1×10^4^ RM1^Luci^ cells were intracardially injected into C57/B6 mice. To monitor the occurrence of metastasis, mice were termly anesthetized and intraperitoneal injected with D-Luciferin (150 mg/kg body weight) for 10 min, and then imaged by IVIS Spectrum Imaging System (Caliper Life Sciences, MA, USA). Organs exhibiting signal of metastasis were harvested and further confirmed by ex vivo imaging. To acquire lung and bone metastatic tendency cells, metastatic lesions tissue was aseptically dissected, digested in 0.25% trypsin and resuspended in complete RPIM 1640 medium. Cells were cultured and reinjected into mice for next round of isolation.

For analyzing the effect of GALNT12 on bone metastatic tendency of PCa, 1×10^4^ GALNT12 knockdown or overexpressed RM1 cells were intracardially injected into C57/B6 mice and monitor the occurrence of metastasis by IVIS Spectrum Imaging System.

For analyzing the immune cells in bone metastatic lesion, intraosseous injection with cells (control and shGALNT12 or GALNT12-WT, 5×10^4^ cells) was performed and the lesion size was monitored by luminescence after 2 weeks. For analyzing the effect of LDN193189 (MCE, New Jersey, USA) on the immune cells in bone metastatic lesion, mice were daily intraperitoneal injected with LDN193189 (3 mg/kg body weight) for 10 days. All animal experiments were performed according to the guidelines of the Ethics Committee of Animal Research of Nanjing Drum Tower Hospital.

### Vicia villosa lectin (VVA) pull-down assay

Cells were lysed using lysis buffer (Byeotime, Shanghai, China) containing phosphatase inhibitor and protease inhibitor. A volume of 200 μL lysate containing 1 mg total protein and 40 μL agarose bound VVA (Vector labs, Newak, USA) were mixed and incubated in rotator at 4 ℃ for 16 h. After that, protein-agarose bound VVA mixture was washed five times using lysis buffer without inhibitor. After centrifugation, 50 μL 2×SDS loading buffer was added to the agarose and denatured by boiling at 95 ℃ for 5 min. The target protein was detected by WB assay.

### Cell adhesion assays

1×10^5^ shNC and shGALNT12 RM1 cells with or without Cyclo(-RGDfK)TFA ((MCE, New Jersey, USA)) were planked into vitronectin (10 μg/mL) (MCE, New Jersey, USA) coated 24-well plate. After incubation at 37°C for 5 min, the plate was gently washed three times with PBS to remove the cells which were not adhered. The remaining adhered cells were cultured in complete medium for 1h. Then cells were fixed with PFA and stained with crystal violet and imaged with microscope.

### Immune cells analysis

Bone metastatic lesions were cut into small fragments and digested with RPMI-1640 medium containing 1.0 mg/mL collagenase type Ⅰ, 250 μg/mL hyaluronidase and 100 U/mL DNase I (Vazyme Biotech, Nanjing, China) for 45 minutes at 37°C to generate tumor mononuclear cells (MNCs). Single-cell suspension was generated though a 70-μm cell strainer and then centrifuged at 400 ×g for 10 min and resuspended with 40% Percoll (Cytiva, Washington D.C., USA). After that, the cell suspension was slowly overlaid on 70% Percoll solution and centrifuged at 400 ×g for 20 minutes. Finally, the obtained cells in the intermediate layer were regarded as MNCs. These cells were washed with PBS and incubated with antibody for 30 min before flow cytometry analysis. The information of antibodies used in this study was list in **Table S2.** The antibody for markers of different immune cells were listed in **Table S3.**

### Co-culture assay

A separated co-culture assay was performed using transwell inserts (0.4 μm, BIOFIL, Guangzhou, China). For co-culture assay with RM1 cells and bone marrow cells or OP-9 cells, RM1 cells were cultured in a 12-well plate, bone marrow cells or OP-9 cells were seeded into upper chambers (0.4 μm, BIOFIL, Guangzhou, China). The co-culture system was maintained for 72h. For co-culture assay with RM1 cells and RAW264.7 cells, RAW264.7 cells were cultured in the wells and RM1 cells were cultured in the upper chambers for 72 h.

### Statistical analysis

GraphPad Prism 8 and IBM SPSS Statistics 17.0 were used for data analysis. All the data are expressed as the mean ± SD. Student t tests was used for independent samples. The repeated measurement data were analyzed by the ANOVA analysis. Kaplan-Meier Plotter was applied for prognostic analysis. Linear correlation analysis was used to assess correlations between GALNT12 expression, integrin αVβ3 expression and the M2 macrophage count. *p*<0.05 was considered statistically significant.

## Results

### Establishment of PCa cells with high bone metastasis tendency

**Figure 1:**
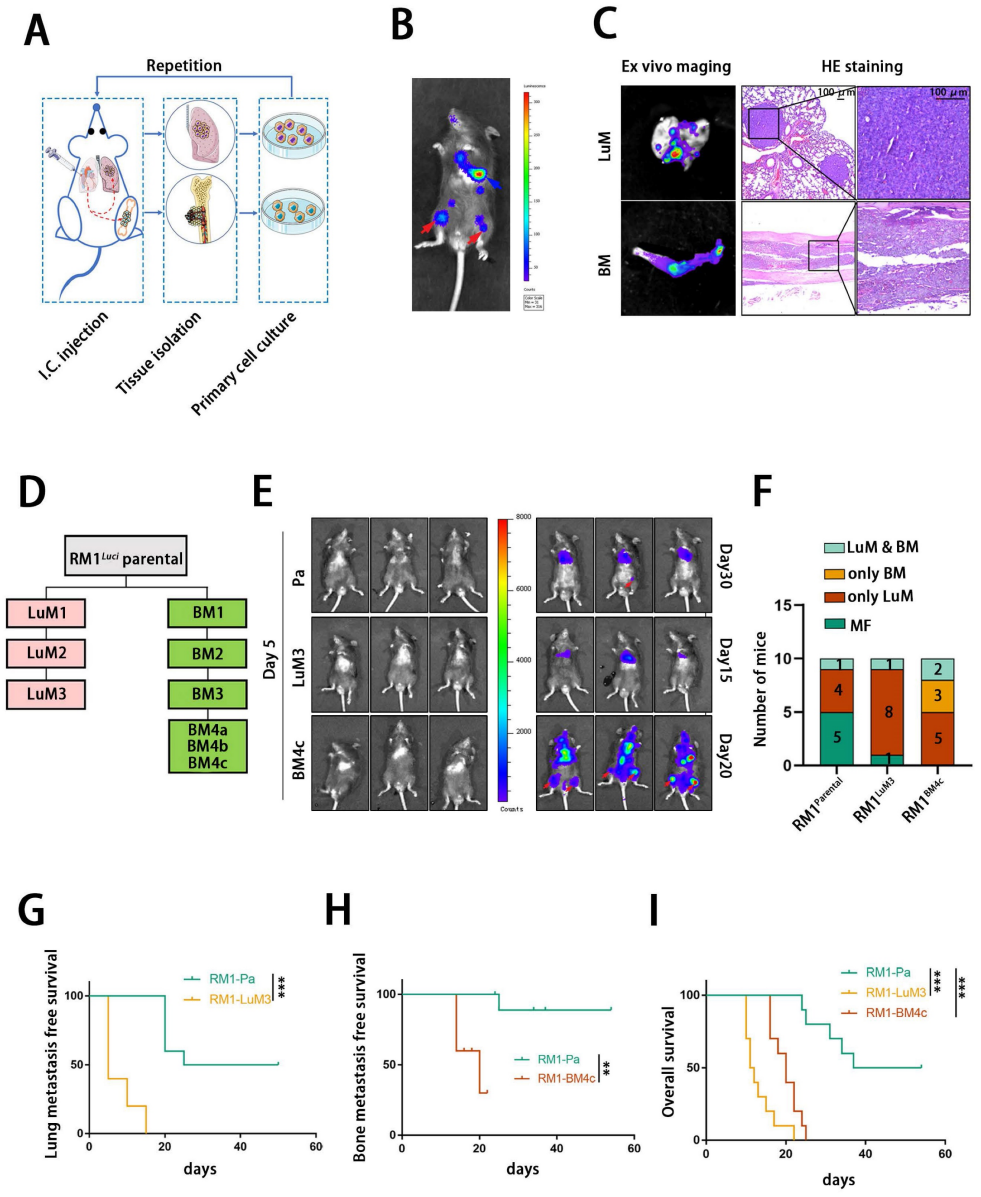
** Isolation, characterization and analysis of bone metastatic derivatives.** A: Construction of the multi-organ metastasis model and screening of organ-specific metastatic derivatives. I.C., intracardiac. B: Representative in vivo bioluminescent image of different organic metastatic lesion, lung metastasis (blue arrow), bone metastasis (red arrow). C: Representative ex vivo bioluminescent and hematein-eosin (H&E) images of lung and bone metastasis, scale bar, 100 μm. D: Flowchart of the selection of lung- and bone-specific metastatic derivatives from RM1^luci^ cells. E: Representative in vivo bioluminescent image of organ metastasis after I.C., injection of different RM1 derivatives (RM1^parental^, n=10; RM1^luM3^, n=10; RM1^BM4c^, n=10) F: statistics of the organic metastasis in different groups respectively for E, MF, metastasis free. G-I: Kaplan-Meier survival curves of RM1^parental^, RM1^LuM3^ and RM1^BM4c^ cells for lung metastasis-free survival (G), bone metastasis-free survival (H) and overall survival (I), respectively. **, *p*<0.01; ***, *p*<0.001.

We firstly constructed organ-specific metastatic derivatives of PCa by metastatic clones isolation, expansion and repeated intracardiac injection using a luciferase-tagged murine PCa cell line RM1^luci^ (**Fig. 1A**). As shown in **Fig. 1B and C**, RM1^luci^ cells mainly metastasized to lung and bone. We named these metastatic derivatives after their source organ and generation. For example, bone metastatic derivative-4c, RM1^BM4c^, is a bone metastatic derivative after the fourth round of selection (**Fig.1 D**). After several rounds of selection, RM1^LuM3^ and RM1^BM4c^ cells exhibited significant metastatic tendency to lung and bone, respectively (**Fig. 1E**). As shown in **Fig. 1F**, the bone-met derivative RM1^BM4c^ showed obviously stronger tendency to bone with 5/10 bone metastasis ratio, compared to 1/10 in RM1^parental^ group and 1/10 in RM1^LuM3^ group, Lung-metastatic derivative RM1^LuM3^ also showed stronger tendency to lung with 9/10 lung metastasis ratio, compared to 5/10 in RM1^parental^ group and 7/10 in RM1^BM4c^ group (**Fig. 1F**). Survival curve analysis also confirmed that mice in RM1^LuM3^ and RM1^BM4c^ group had shorter lung and bone metastasis-free survival than RM1^parental^ group, repectively (**Fig.1 G-H**). Compared to the parental group, the overall survival (OS) of RM1^LuM3^ and RM1^BM4c^ groups were also significantly worse (**Fig. 1I**).

### GALNT12 was a potential regulator in bone metastasis of PCa

To analyze key genes specifically involved in bone metastasis of PCa, RNA sequencing was performed in RM1^parental^, RM1^LuM3^, and RM1^BM4c^ cells. The transcription profiles were significantly distinct among these three groups (**Fig. S1A-C**). KEGG analysis and GO analysis showed that RM1^LuM3^ and RM1^BM4c^ cells exhibited significantly different profile (**Fig. S1D-E**). For example, KRGG analysis showed that the most changed genes in RM1^LuM3^ cells were associated with “focal adhesion” while such genes in RM1^BM4c^ cells were associated with “natural killer cell mediated cytotoxicity” (**Fig. S1D**). In addition, we further analyzed an independent data from GEO DataSets (GSE32269), which compared differently expressed genes between clinical primary PCa tissues and bone metastasis 20.

**Figure 2:**
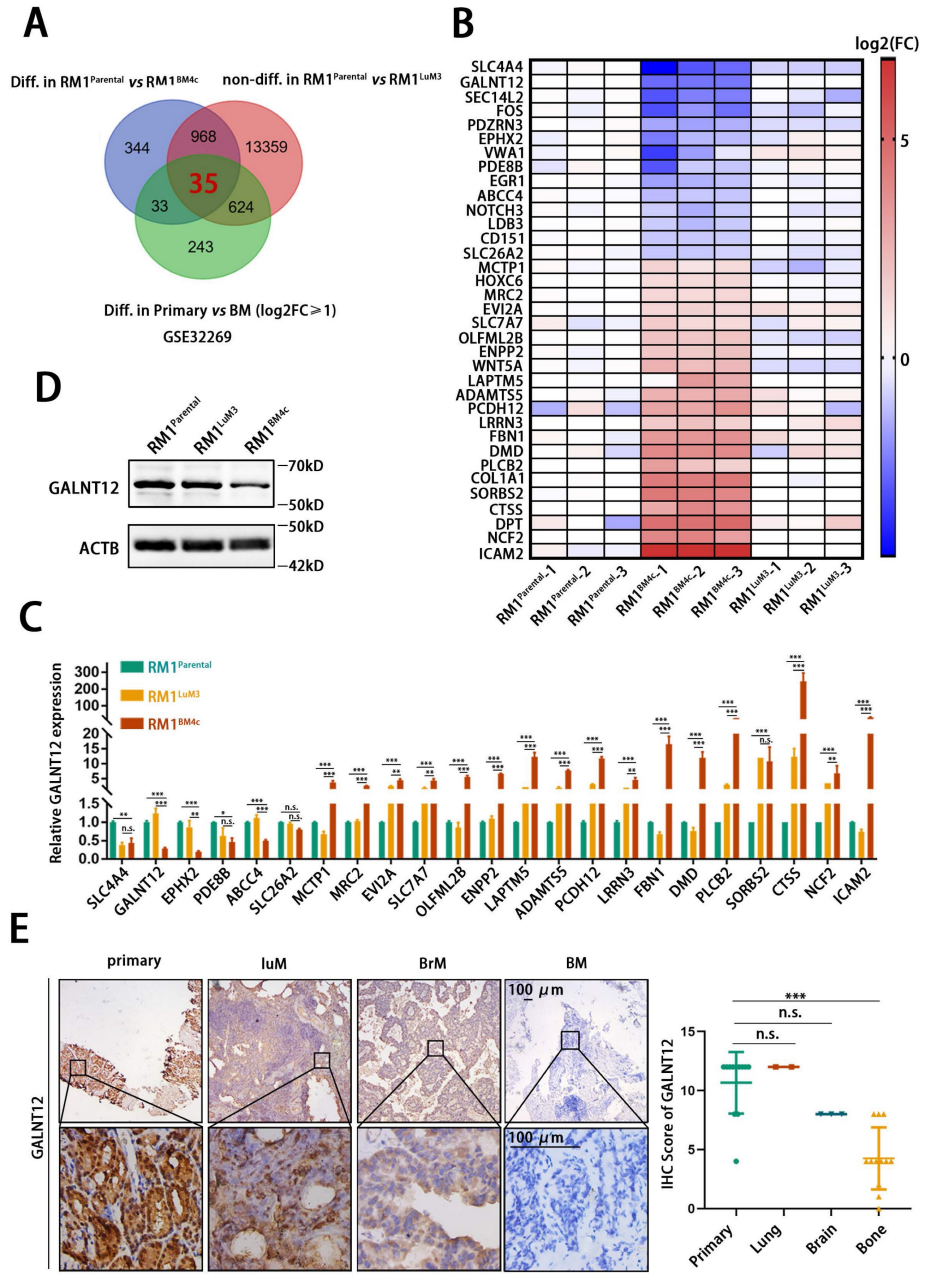
**GALNT12 was downregulated in bone metastasis of prostate cancer.** A: Genes which were differentially expressed in RM1^BM4c^/RM1^parental^, bone-metastatic lesion/primary lesion and not differentially expressed in RM1^LuM3^/RM1^parental^ were analyzed by Venn analysis. B: Heatmap showing the expression of 35 genes in RM1^BM4c^ and RM1^LuM3^ cells compared to RM1^parental^. C: The expression of 23 out of the 35 genes was measured by qPCR. D: The expression of GALNT12 in RM1^parental^, RM1^LuM3^ and RM1^BM4c^ was measured by WB. E: Representative IHC images for GALNT12 in clinical samples of primary PCa tissue, lung metastasis (LuM), brain metastasis (BrM) and bone metastasis (BM), scale bar, 100 μm (left panel) and quantitation of staining score (right panel). n.s., no significant; *, *p*<0.05; **, *p*<0.01; ***, *p*<0.001.

Integrating analysis of these two datasets, we finally identified 35 genes (21 up-regulated and 14 down-regulated) that might specifically were involved in bone metastasis of PCa (**Fig. 2A-B**). These 35 genes were significantly altered in RM1^parental^ *vs* RM1^BM4c^, and Primary *vs* BM, but unchanged in RM1^parental^ *vs* RM1^LuM3^. Considering their potential functions in tumor progression and existing research status, we screened 23 candidate genes to furtherly verify their expression in RM1^parental^, RM1^LuM3^, and RM1^BM4c^ cells by RT-qPCR (**Fig. 2C, Fig. S2A**). These results found that polypeptide N-acetylgalactosaminyltransferase 12 (GALNT12), a glycosylase associated with O-glycosylation, was the most down-regulated gene in the comparison of RM1^BM4c^ to RM1^parental^ and RM1^BM4c^ to RM1^LuM3^ cells (**Fig. 2C**), and showed unchanged between RM1^parental^ and RM1^LuM3^ cells. Reduced GALNT12 protein level was also confirmed in RM1^BM4c^ cells (**Fig. 2D, Fig. S2B**). We furtherly validated the result in different metastatic tissues of PCa patients by IHC. As expected, GALNT12 was specifically decreased in bone metastases rather than lung and brain metastases compared to that in primary PCa tissues (**Fig. 2E**). Taken together, GALNT12 might be a crucial regulator in bone metastasis of PCa.

### GALNT12 depletion promoted bone metastasis of PCa

**Figure 3:**
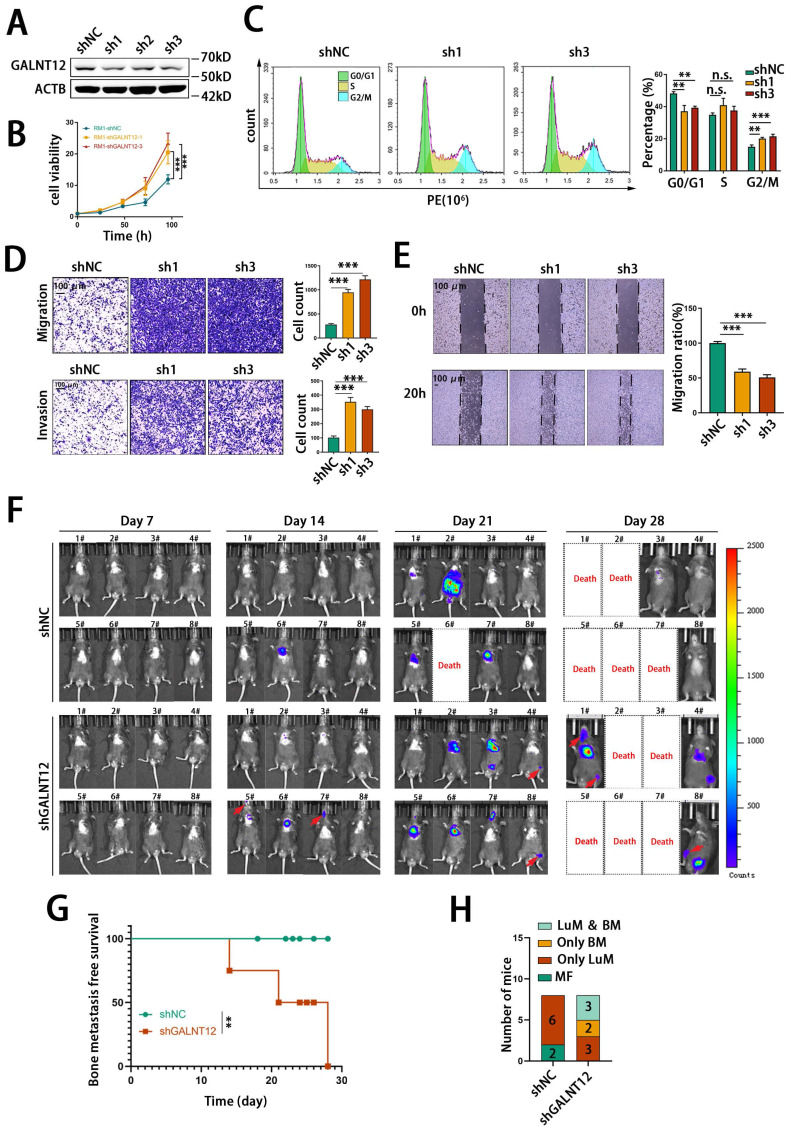
**GALNT12 suppressed the metastasis of PCa *in vitro* and *in vivo*.** A: knockdown efficiency of shRNA for GALNT12 in RM1 cells was verified by WB. B: MTT assay of control and GALNT12-knockdown RM1 cells for proliferation. C: Flow cytometry assay for cell cycle (left panel) and quantitation of proportion of cell cycle (right panel). D: Transwell assay of shNC and shGALNT12 RM1 cells for migration (upper panel) and invasion (lower panel) and quantitation of the ratio respectively (right panel), scale bar, 100 μm. E: Wound heal assay of shNC and shGALNT12 RM1 cells for migration (left panel) and quantitation of the migration ratio (left panel), scale bar, 100 μm. F: In vivo bioluminescent images of organic metastasis after I.C., injection of shNC and shGALNT12 RM1 cells. G: Kaplan-Meier survival curves of shNC and shGALNT12 cells, MFS, metastasis-free survival. H: Statistics of the organic metastasis in different groups respectively. n.s., no significant; **, *p*<0.01; ***, *p*<0.001.

In order to evaluate the role of GALNT12 in bone metastasis of PCa, we firstly knocked down the expression of GALNT12 in RM1 cells using shRNAs (**Fig. S2C, Fig. 3A**). Considering GALNT12 depletion efficiency, sh1 and sh3 were chosen for further study. As shown in **Fig. 3B**, GALNT12 deficiency could significantly increase cell proliferation ability and cell cycle analysis showed obviously lower proportion of cells at G0/G1 phase and higher proportion of cells at G2/M phase when knocking down GALNT12 in RM1 cells (**Fig. 3C**). Transwell assay and wound healing assay indicated that depletion of GALNT12 could greatly enhanced the migration and invasion abilities of PCa cells (**Fig. 3D-E**). However, GALNT12 did not seem to affect the function of PCa cells on sphere formation, clone formation, apoptosis, and anoikis resistance (**Fig. S2D-G**).

We further intracardially injected GALNT12-depleted RM1 cells in mice to assess their metastatic potential *in vivo*. The results indicated that knocking down GALNT12 in RM1 cells significantly increased the bone metastasis ratio in mice (**Fig. 3F-G**), while had little effect on lung metastasis (**Fig. 3H**). Together, these results demonstrated that depletion of GALNT12 in PCa cells significantly enhanced the proliferation, migration and invasion abilities of PCa cells and promoted high bone metastasis of PCa.

### The glycosylase function is crucial for the role of GALNT12 in PCa

**Figure 4:**
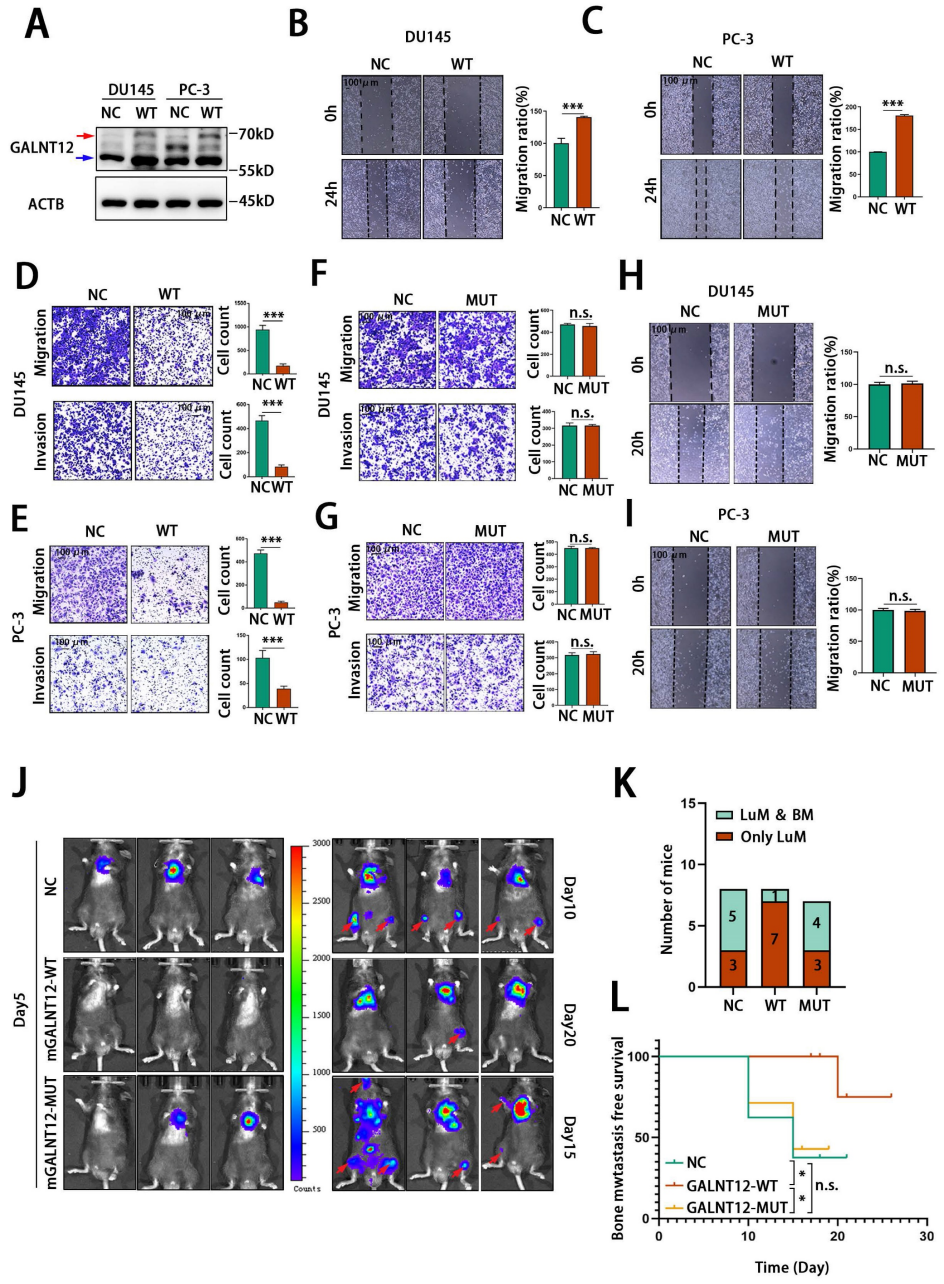
**GALNT12 suppressed the metastasis of PCa depending on its enzymatic activity.** A: The efficiency of GALNT12-overexpressed vector was measured by WB. B-C: Wound heal assay of control and GALNT12-WT DU145 (B) and PC-3 (C) cells (left panel) and quantitation of the migration ratio (right panel), scale bar, 100 μm. D-E: Transwell assay of control and GALNT12-WT DU145 (D) and PC-3 (E) cells for migration (upper panel) and invasion (lower panel) and quantitation of the invaded ratio respectively (right panel), scale bar, 100 μm. F-G: Transwell assay of control and GALNT12-MUT DU145 (F) and PC-3 (G) cells for migration (upper panel) and invasion (lower panel) and quantitation of the invaded ratio respectively (right panel), scale bar, 100 μm. H-I: Wound heal assay of control and GALNT12-MUT DU145 (H) and PC-3 (I) cells for migration (upper panel) and quantitation of the migration ratio (lower panel), scale bar, 100 μm. J: In vivo bioluminescent images of organic metastasis after I.C., injection of NC (day10), mGALNT12-WT (day20) and mGALNT12-MUT (day15) RM1 cells. K: Statistics of the organic metastasis in different groups respectively. L: Kaplan-Meier survival curves of NC, GALNT12-WT and GALNT12-MUT RM1 cells. n.s., no significant; ***, *p*<0.001. Red arrow: exogenous GALNT12; Blue arrow: endogenous GALNT12.

**Figure 5:**
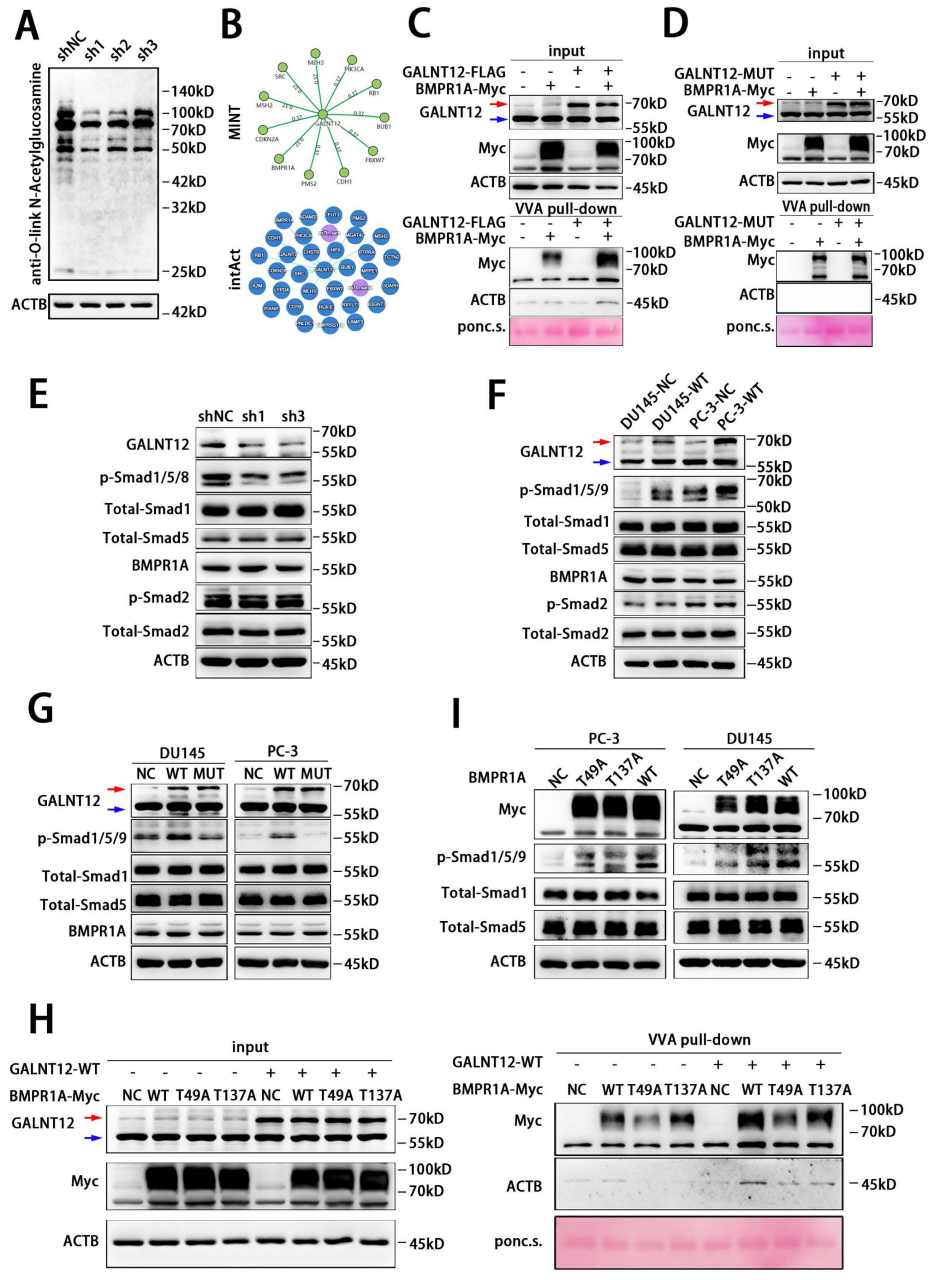
** GALNT12 activated BMP signaling via promoting the O-GalNAcylation of BMPR1A.** A: The O-glycosylation of shGALNT12 RM1 cells was measured by WB. B: The potential proteins modified by GALNT12 were predicated by MINT and intAct websites. C: GALNT12 stable expressed HEK 293T cells were transfected with BMPR1A expression vectors and subjected to pull-down with VVA-agarose for evaluating the O-GalNAcylation of BMPR1A. D: VVA pull-down assay for the effect of enzymatic activity of GALNT12 on the O-GalNAcylation of BMPR1A. E-F: effect of GALNT12 expression on BMP and TGFβ signaling in mouse PCa cells (E) and human PCa cells (F). G: Effect of enzymatic activity of GALNT12 on BMP signaling. H: The effect of mutant sites of BMPR1A for the BMP signaling. I: VVA pull-down assay for the modified sites of BMPR1A for GALNT12. Red arrow: exogenous GALNT12; Blue arrow: endogenous GALNT12.

As a glycosylase, GALNT12 promotes the O-GalNAcylation in the initial step of O-linked protein glycosylation. To evaluate whether GALNT12 exerted its effects on bone metastasis of PCa through the glycosylase function, we constructed a human GALNT12 overexpression plasmid (hGALNT12-WT) (**Fig. S3B and Fig. 4A**) and a corresponding plasmid with mutation in the catalytic structural domain of GALNT12 (hGALNT12-MUT) (**Fig. S3C and Fig. 5H**). We observed that the band of exogenous GALNT12 was larger than endogenous GALNT12 and that might be caused by the additional tags of overexpressed plasmid. As shown in **Fig. 4B-E**, overexpression of GALNT12 in GALNT12 low-expressed human PCa cells (DU145 and PC-3) (**Fig. S3A**) significantly reduced the migration and invasion abilities of PCa cells and suppressed the proliferation ability of PCa cells (**Fig. S3D-G**), while mutation in catalytic structural domain of GALNT12 could completely remove its suppression effects on migration and invasion abilities of PCa cells (**Fig. 4F-I**). We further restored GALNT12 expression in RM1^BM4c^ cells with mouse GALNT12-WT (mGALNT12-WT) or GALNT12-MUT (mGALNT12-MUT) (**Fig. S3H-I**) lentivirus, and then intracardially injected these cells in mice. These results showed that mGALNT12-WT could obviously reduce the bone metastasis tendency of RM1^BM4c^ cells, while mGALNT12-MUT had no that effect (**Fig. 4J-L**). Thus, these findings suggested that the glycosylase function of GALNT12 was crucial for its role in PCa.

### GALNT12 mediated the O-GalNAcylation of BMPR1A to activate BMP signaling

Knocking down GALNT12 decreased the total protein O-GalNAcylation level in RM1 cells (**Fig. 5A**). We next predicted potential targets of GALNT12 via The Molecular INTeraction Database (https://mint.bio.uniroma2.it/) and IntAct Molecular Interaction Database (https://www.ebi.ac.uk/intact) (**Fig. 5B**), and found that bone morphogenetic protein receptor type 1A (BMPR1A) was the common predicted target between the two database. VVA-pull down assay was further performed to evaluate whether GALNT12 directly mediated the O-GalNAcylation of BMPR1A. We observed that the O-GalNAcylation level of BMPR1A was markedly increased in cells co-expression with hGALNT12-WT (**Fig. 5C**), while had no change in cells co-expression with hGALNT12-MUT (**Fig. 5D**).

BMPR1A is one of the key receptors of BMP signaling pathway. Intriguingly, BMP signaling pathway has been reported to be crucial in bone metastasis of PCa^21^, and exerted its effects via downstream mediator SMAD proteins (phosphorylation of Smad1/5/9 in human and Smad1/5/8 in mice).

To investigate the effect of GALNT12 on BMP signaling, we knocked down GALNT12 in RM1 cells and found a significant decreased phosphorylation of Smad1/5/8 (**Fig. 5E**). And overexpression of hGALNT12-WT in DU145 and PC-3 cells obviously accelerated the phosphorylation of Smad1/5/9 (**Fig. 5F**), when overexpressing hGALNT12-MUT in PCa cells, the phosphorylation level of Smad1/5/9 had no change (**Fig. 5G**). Moreover, neither the expression level nor the glycosylase function mutation of GALNT12 had effect on the protein level of BMPR1A (**Fig. 5E-G**), suggesting that GALNT12 might activate the BMP pathway by promoting the enzymatic activity rather than the protein level of BMPR1A. To determine the specific O-GalNAcylation sites of BMPR1A, we firstly predicted the potential sites via ISOGlyp website (https://isoglyp.utep.edu/), and found that Thr^49^ and Thr^137^ in BMPR1A were more likely to undergo O-GalNAcylation (**Fig. S3J**). Whereafter, site-directed mutagenesis was preformed to replace threonine residues with alanine (**Fig. S3J**). In VVA-pull down assays, we found that both Thr^49^ and Thr^137^ mutation could obviously reduce the O-GalNAcylation level of BMPR1A (**Fig. 5H**). These results indicated that GALNT12 directly mediated the O-GalNAcylation of BMPR1A at Thr^49^ and Thr^137^ sites. Furthermore, we over-expressed Thr^49^ mutation BMPR1A, Thr^137^ mutation BMPR1A, or wild BMPR1A in PCa cells, and found that p-Smad1/5/9 levels in T49A and T137A cells were both increased compared to that in control cells, but still lower than that in cells over-expressing wild BMPR1A (**Fig. 5I**). These results indicated that Thr^49^ and Thr^137^ sites both contributed to the function of BMPR1A on mediating BMP signaling. The crosstalk between BMP and TGFβ pathway was observed before 22. Thus, we examined whether GALNT12 exerts its metastasis promoting effect on PCa through TGFβ pathway. In contrast to the effect on BMP signaling, expression of GALNT12 had no effect on the TGFβ-induced Smad2 phosphorylation (**Fig. 5 E-G**).

**Figure 6:**
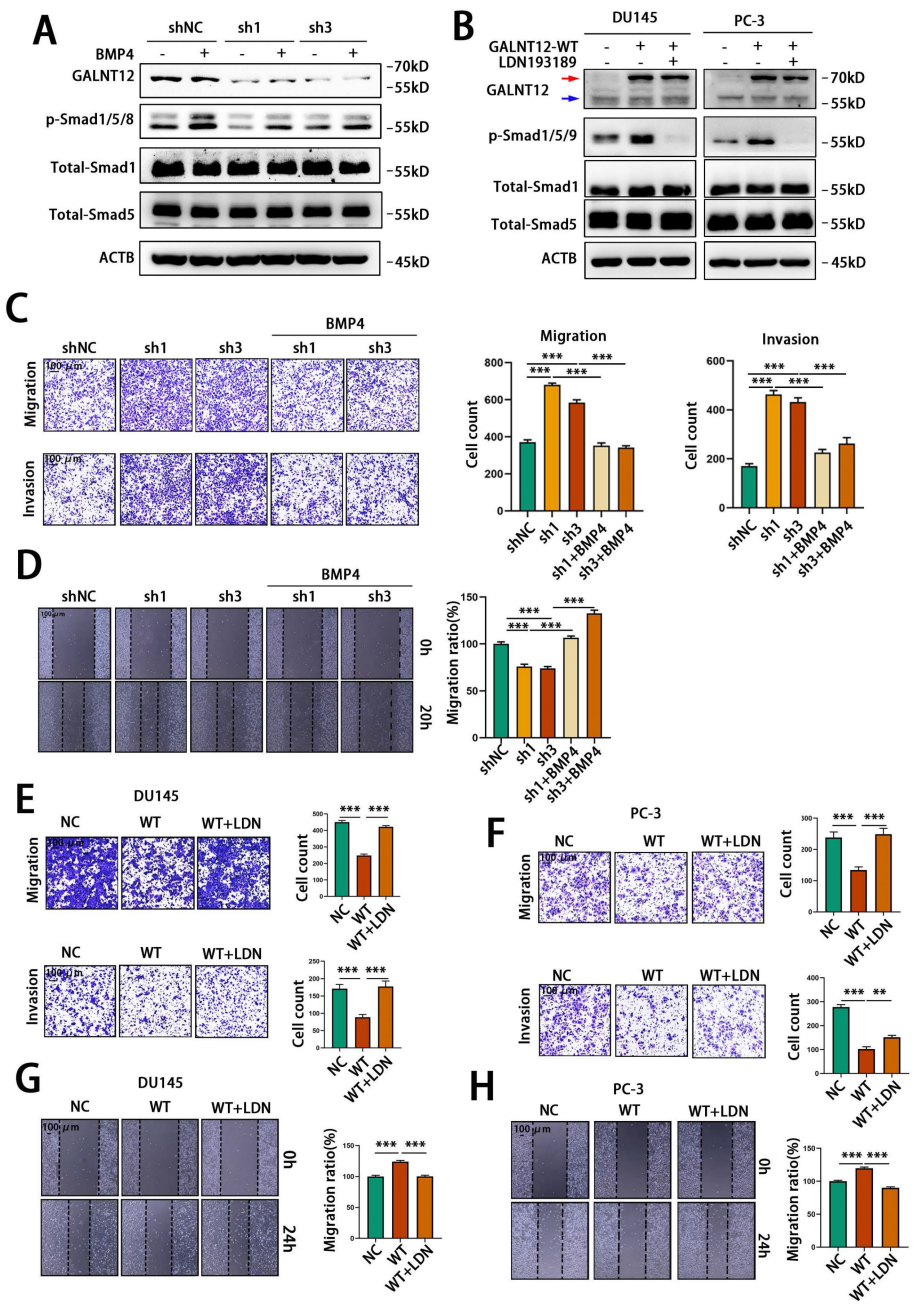
** GALNT12 suppressed metastasis of PCa cells by activating BMP4 signaling.** A: WB analysis of control and shGALNT12 RM1 cells treated with BMP4 (3ng/ml) for the effect on BMP signaling. B: WB analysis of control and GALNT12-WT DU145 and PC-3 cells treated with LDN193189 for the effect on BMP signaling. C: Transwell assay of control and shGALNT12 RM1 cells with and without BMP4 treated for migration (upper panel) and invasion (lower panel) and quantitation of the invaded ratio respectively (right panel). D: Wound heal assay of control and shGALNT12 RM1 cells with and without BMP4 treatment for migration (left panel) and quantitation of the migration ratio respectively (right panel). E-F: Transwell assay of control and GALNT12-WT DU145 (E) and PC-3 (F) cells with or without LDN193189 treatment for migration (upper panel) and invasion (lower panel) and quantitation of the invaded ratio respectively (right panel). G-H: Wound heal assay of control and GALNT12-WT DU145 (G) and PC-3 (H) cells (left panel) with and without LDN193189 treatment and quantitation of the migration ratio (right panel). **, *p*<0.01; ***, *p*<0.001. Red arrow: exogenous GALNT12; Blue arrow: endogenous GALNT12.

Furthermore, we performed rescue experiments to confirm that GALNT12 activated BMP signaling via BMPR1A. BMP4 had been reported to activate BMP signaling through binding to BMPR1A^23^. We used recombinant murine BMP4 to treat RM1 cells, and observed BPM4 dose-dependent increase of the phosphorylation of Smad1/5/8 (**Fig. S4A**). Treating control or GALNT12-depleted RM1 cells with 3 ng/mL BMP4 for 12 hours demonstrated that knocking down GALNT12 could obviously block the activation of BMP signaling by BMP4 (**Fig. 6A**). In addition, LDN193189 was a BMPR1A inhibitor 24 which could significantly suppress BMP signaling in DU145 and PC-3 cells. We found that treating PCa cells with LDN193189 greatly reduced the promotion effect of GALNT12 on BMP signaling (**Fig. S4B and Fig. 6B**). Taken together, these results revealed that GALNT12 activated BMP signaling in PCa cells through mediating O-GalNAcylation of BMPR1A.

### GALNT12-BMP signaling suppressed bone metastasis of PCa via modulating integrin αVβ3 expression

Firstly, to investigate whether GALNT12 suppress the migratory and invasive abilities of PCa cells by activating BMP signaling, BMP4 was used to treat control or GALNT12-depleted RM1 cells. As shown in **Fig. 6C** and** 6D**, BMP4 could reverse the promotive effect of GALNT12 depletion on the migratory and invasive abilities of PCa cells to a certain extent. Furthermore, treating DU145 and PC-3 cells with LDN193189 greatly restored the migratory and invasive abilities reduced by GALNT12 overexpression in PCa cells (**Fig. 6E-H**). Thus we concluded that GALNT12 suppressed the migratory and invasive abilities of PCa cells by activating BMP signaling.

**Figure 7:**
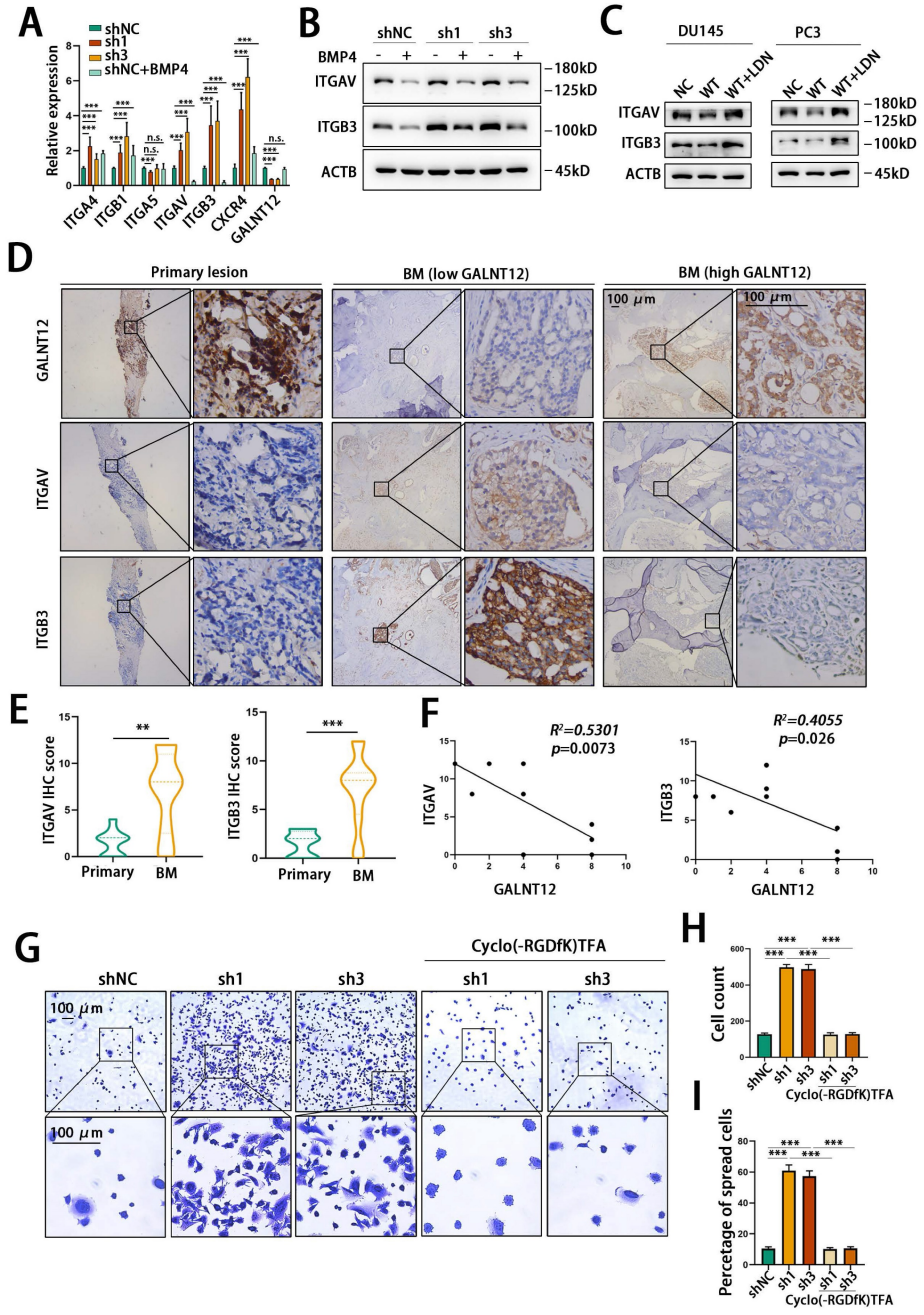
**GALNT12 suppressed bone-specific metastasis of PCa via regulating the expression of integrin αVβ3.** A: The expression of adhesion molecules in shGALNT12 and BMP4 treating RM1 cells was measured by qPCR. B: WB analysis of control and shGALNT12 RM1 cells treated with BMP4 (3ng/ml) for the effect on the expression of integrin αVβ3. C: WB analysis of control and GALNT12-WT DU145 and PC-3 cells treated with LDN193189 for the effect on the expression of integrin αVβ3. D: Representative IHC staining images for integrin αVβ3 expression in GALNT12^low^ and GALNT12^high^ bone metastatic lesions, respectively. E: Quantitation of integrin αVβ3 expression in GALNT12^low^ and GALNT12^high^ bone metastatic lesions F: The correlation analysis between the expression of GALNT12 and integrin αVβ3. G-I: Representative crystal violet staining images for the adhered ability of control and shGALNT12 RM1 cells with and without BMP4 treating (G) and quantitation of the adhered cells (H) and the spread cells(I). n.s., no significant; **, *p*<0.01; ***, *p*<0.001.

However, it was still not clear how GALNT12 depletion promoted high bone metastasis tendency of PCa. As reported, the unique advantage of seeding cells in colonizing target organ was crucial for organ-specific metastasis 25. Integrins are heterodimeric transmembrane glycoproteins, they mediate cell to extracellular matrix (ECM) and cell to cell adhesion and migration. Numerous studies demonstrated that cancer cells localized to specific organ tissues via integrin-mediated contacts with ECM and stromal cells, and alteration of integrin expression and signaling in tumor cells led them to hold the abilities to escape from original context, and colonize target tissues 26. Integrin α4β1, αVβ3 and α5β1 and CXCR4 played key roles during bone metastasis of PCa 27. To investigate whether GALNT12-BMP signaling suppress bone metastasis via alteration of these integrins, we detected mRNA levels of these intergrins in GALNT12-depleted or BMP4 treated RM1 cells. As shown in **Fig. 7A**, ITGAV and ITGB3 were upregulated in GALNT12-depleted cells, while downregulated in cells treated with BMP4. ITGAV and ITGB3 protein levels were also increased in GALNT12-depleted RM1 cells and reversed after treating with BMP4 (**Fig. 7B**). Over-expressing GALNT12 in DU145 and PC-3 cells led to decrease expression of ITGAV and ITGB3, and they could be further restored with LDN193189 treatment (**Fig. 7C**). In clinical PCa tissues, ITGAV and ITGB3 were obviously enriched in bone metastasis compared to that in primary PCa tissues, and showed significantly negative correlation with levels of GALNT12 in PCa bone metastasis (**Fig. 7D-F**). These results indicated that the expression of ITGAV and ITGB3 were modulated by GALNT12-BMP signaling.

ITGAV and ITGB3 encoded intergrin αV and intergrin β3 respectively, and they constituted the α and β subunits of integrin αVβ3. αVβ3 had been identified as a crucial integrin promoting bone metastasis of PCa via binding to the vitronectin which was abundantly expressed in bone matrix 28. We then assessed the effect of GALNT12 depletion on the ability of RM1 cells to adhere to and migrate on vitronectin. We found that knocking down GALNT12 obviously promoted RM1 cells to adhere to plate (**Fig. S4C**) and also induced a greatly increased counts of cells adhering and spreading to vitronectin (**Fig. 7G-I**). And treating RM1 cells with Cyclo(-RGDfK)TFA, an inhibitor of αVβ3, could obviously block the promotion effects of GALNT12 depletion on the ability of cells binding to the vitronectin (**Fig. 7G-I**). Therefore, these results revealed that GALNT12-BMP signaling suppressed bone specific metastasis of PCa via modulating integrin αVβ3 expression.

Androgen receptor (AR) was considered extremely important in the progression of PCa^29^. We then assessed the GALNT12-BMPR1A-integrin αVβ3 axis in Lncap cells, an AR-positive cell line. We found that knocking down GALNT12 also promoted the expression of ITGAV and ITGB3 via BMP signaling in Lncap cells. What's more GALNT12 depletion could upregulate the expression of KLK3 which encodes the prostate-specific antigen (PSA) protein, suggesting the activation of AR signaling after GALNT12 depletion (Fig. S3J). Consistent with this result, there was also a study reporting that the phosphorylation of Smad1 could induce the interaction between Smad1 and AR, thus inhibited the AR-mediating transactivation 30. However, treating Lncap cells with a synthetic androgen R1881 showed little effect on the expression of GALNT12 (Fig. S3K).

### GALNT12-BMP signaling modulated the immune microenvironment of bone metastasis via suppressing STAT3 signaling

**Figure 8:**
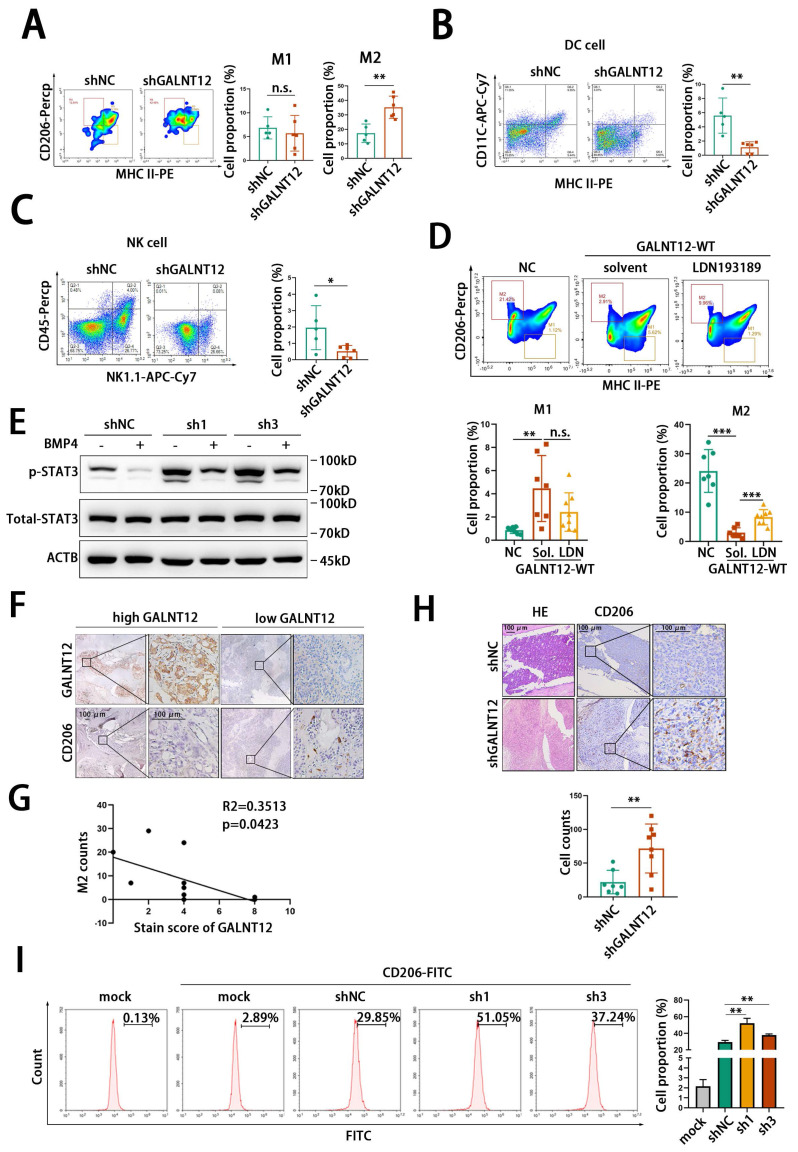
** GALNT12 remolds the immune microenvironment of bone-metastatic lesion of PCa.** A-C: Representative flow cytometry analysis images for proportion of DC cells (A), NK cells (B) and macrophage (C) in mouse bone metastatic lesion (left panel) and quantitation of cells proportion (right panel). D: Representative flow cytometry analysis images for proportion of M2 macrophage cells in GALNT12-WT overexpressed RM1^BM4c^ cells with or without LDN193189 treatment (upper panel) and quantitation of cells proportion (lower panel). E: WB analysis of the effect of shGALNT12 and BMP4 treating on STAT3 signaling. F: Representative IHC staining of M2 macrophage in human bone metastatic lesion, scale bar, 100 μm. G: The correlation analysis between M2 macrophage count and expression of GALNT12. H: Representative HE staining images and IHC staining of M2 macrophage in mouse bone metastatic lesion (left panel) and quantitation of M2 macrophage cells count (right panel), scale bar, 100 μm. I: Representative flow cytometry analysis images for proportion of CD206^+^ RAW264.7 cells (left panel) and quantitation of CD206^+^ RAW264.7 cells (right panel). n.s., no significant; *, *p*<0.05; **, *p*<0.01; ***, *p*<0.001.

Bone microenvironment is a special microenvironment with unique bone marrow niches and myeloid cells contributing to metastasis, colonization, dormancy, activation, and immune escape of cancer cells. Remodeling bone immune microenvironment has been reported to be an important way for bone metastasis of PCa. Therefore, to investigate whether GALNT12 was involved in bone immune microenvironment remodeling, we performed intraosseous injection with stably GALNT12-depleted RM1 cells in mice. We found that tumor development in bone was significantly promoted in GALNT12 depletion group compared to that in control group. Further analyzing the proportion of multiple immune cells in tumors showed that GALNT12 knockdown could promote the infiltration of M2 macrophages and suppress the infiltration of dendritic cells (DC) and natural killer (NK) cells in bone metastatic lesions (**Fig. 8A-C**), while showed no effect on the infiltration of T cells, B cells, myeloid-derived suppressor cells (MDSC), neutrophil and regulatory T (Treg) cells (**Fig. S4D-H**).

To investigate whether GALNT12 modulated the immune microenvironment through BMP signaling, GALNT12 overexpressing RM1 cells were intraosseously injected in mice and treated with LDN193189 and the weight of mice was monitored during the LDN193189 treatment (**Fig. S4L**). We found that GALNT12 overexpression suppressed the infiltration of M2 macrophages and promoted the infiltration of NK cells and DC cells in bone metastatic lesions and their alteration could be reversed after treated with LDN193189(**Fig. 8D, Fig. S4I-J**).

STAT3 was reported to be strongly associated with the infiltration of M2, DC and NK cells in immune microenvironment, as reported, activating STAT3 signaling could induce the infiltration of M2 macrophages 31, 32 and suppress the infiltration of DC 33 and NK 34 cells. We found that knocking down GALNT12 could activate STAT3 signaling and treating with BMP4 could markedly block the stimulative effect of GALNT12 depletion on STAT3 signaling (**Fig. 8E**).

Comparing to DC and NK cells, M2 macrophages were the most recognized immune cell type contributing to the immunosuppressive microenvironment of bone metastases in multiple cancers including PCa 35, 36. In the present study, we further demonstrated that the protein levels of GALNT12 was significantly negatively correlated with the enrichment of M2 macrophages in bone metastases of PCa patients (**Fig. 8F-G**) and mouse model (**Fig. 8H**). And co-cultured RAW264.7 with GALNT12 depleted RM1 cells could obviously promote the polarization of M2 macrophages (**Fig. 8I**). Surprisingly, we also found that the protein levels of GALNT12 in RM1 cell were significantly decreased after co-culturing with bone marrow or OP-9 cells (**Fig. S4K**). And treating RM1 cells with TGFβ1, a crucial cytokine of M2 macrophages, could also obviously reduce GALNT12 levels (**Fig. S4K**). These results suggested that a feedback regulation mechanism might exist to amplify the suppressive effects of GALNT12 in PCa cells on bone immune microenvironment, which needed further exploration.

Taken together, we speculated that GALNT12-BMP signaling might modulate the bone immune microenvironment to prevent the survival of PCa cells in bone via suppressing STAT3 signaling.

## Discussion

Bone metastasis is the principal challenge in the treatment of advanced PCa. Despite lots of efforts have been made, it is still unclear to the mechanism by which PCa cells metastasize to bone with high specificity. Bone metastasis is a multi-step and complicated process. During the process, intrinsic migration and invasion abilities of PCa cells, colonization of target organs, and remodeling of the microenvironment in metastatic sites are all events contributing to organ-specific metastasis 12, 15, 16, 23. In our present study, we demonstrated that GALNT12 was a powerful bone metastasis suppressor, which restrained PCa cells from metastasizing to bone by regulating all three of these events concurrently.

GALNT12 is one of the GALNT family members, mediating the O-glycosylation of proteins. Several studies have reported the association between GALNT12 and carcinogenesis in colon cancer 37 and glioblastoma 38. However, the functional role of GALNT12 in PCa development, especially in bone-specific metastasis, is poorly understood. In this study, through integrating analysis of the transcriptional profile of clinical samples and bone/lung-specific metastatic RM1^luci^ derivatives, we identified GALNT12 might be a key suppressor of bone metastasis in PCa. Phenotypically, depletion of GALNT12 contributed to cell proliferation, migration, and invasion of PCa cells *in vitro*, as well as promoted bone metastasis tendency, while had little effect on lung metastasis tendency *in vivo*. Lung metastatic tumor burden was the leading cause of death in mice with metastasis. That might be the reason why GALNT12 depletion reduced the bone-metastasis-free survival but has little effect on OS. Mechanistic research indicated that GALNT12 exerted the suppressive effects on bone metastasis through its glycosylase function, and BMPR1A, one of the key receptors of BMP signaling pathway, was the crucial O-GalNAcylation substrate of GALNT12.

BMP signaling is involved in a wide range of cellular process, and its role in tumor growth and metastasis are controversial. Plentiful of studies have reported that BMPs may either play pro-tumorigenic roles via enhancing cell proliferation, motility, and invasion in cancers or, on the contrary, suppress the metastasis of cancer cells 39. These reports suggest that BMPs function in a context dependent manner, and switch the oncogenic or tumor-suppressive role under certain conditions, however, the underlying mechanism remains unrevealed. Here, we confirmed the suppressor function of BMP signaling in bone-specific metastasis of PCa cells, and demonstrated that GALNT12-mediated O-GalNAcylation of BMPR1A was the activator of BMP signaling in PCa. Supporting this discovery, there was also study reporting that the O-GalNAcylation of BMPR1A by GALNT8 enhanced its activity to transduce BMP signaling in breast cancer 40. In addition, we revealed that integrin αVβ3 and STAT3 signaling were both downstream pathways restrained by BMP signaling in PCa and GALNT12-BMP signaling significantly impeded the adhesion of PCa cells to bone matrix and modulated the immune microenvironment of bone through these two pathways respectively.

Adhesion between circulating tumor cell (CTC) and extracellular matrix (ECM) in metastasis site is a key event leading the organ tropism of metastasis 41. Integrin family is considered to mediate the adhesion between cancer cells and ECM 42, 43. In bone metastasis of PCa, αVβ3 is the most recognized integrin, which can regulate the adhesion and migration of PCa cells via combining with ECM and cell surface ligands 44. Many investigations are performed to explore the regulatory and functional mechanism of αVβ3 during PCa metastasis. For instance, IL-8, IGF-I, and CCN2 are all ligands that can promote the expression of αVβ3 through activating different signaling pathways to elevate the invasion of PCa cells 44. While BKCa and CXCL16 promote the aggregation and activation of αVβ3, thus trigger the activation of downstream FAK to enhance tumor cell migration and invasion abilities 44. In this study, we found ITGAV and ITGB3, genes coding the integrin αVβ3, were regulated by GALNT12-BMP signaling. In clinical bone metastatic tissues of PCa, integrin αVβ3 was also overexpressed and negatively correlated with GALNT12 levels. We further demonstrated that GALNT12-BMP signaling decreased integrin αVβ3 expression, suppressed the binding of cancer cells to the vitronectin in bone matrix, and thus restrained bone metastasis of PCa. In addition to combine with ECM, αVβ3 was also reported to directly promote proliferation, invasion and survival of cancer cells 43. In our present study, we also found that dysregulation of GALNT12 could affect the proliferation, migration and invasion of PCa cells *in vitro*, and that might be mediated or partly mediated via αVβ3 pathway.

The tumor immune microenvironment (TIME) of metastasis niche contributes to the distant metastasis of cancer cells 45. And the distinct immune microenvironment in bone metastases is the crucial obstacle to successfully therapy bone metastatic PCa patients 46. A recent study reported that bone-metastatic PCa was associated with multifaceted immune distortion including T cell exhaustion and M2 macrophage polarization by analyzing single cells from bone marrow of bone metastatic PCa patients 35, 36, suggesting the therapeutic value of targeting immune microenvironment of bone metastases for bone metastatic PCa patients. The KEGG enrichment analysis of our RNA sequencing also showed that genes involved in natural killer cell mediated cytotoxicity and antigen processing and presentation were significantly changed in bone metastasis derivative RM1 cells. Furthermore, we analyzed the proportion of multiple immune cells in bone metastases from mice intraosseously implanted with stably GALNT12-depleted RM1 cells via flow cytometry and found that GALNT12 depletion promoted the M2 macrophages enrichment and suppressed DC and NK cells infiltration, and the alteration could be reversed by the inhibitor of BMP signaling. In summary, our results indicated that the expression of GALNT12 in PCa cells could be reduced by TGFβ1, a potent immunosuppressive cytokine of M2 macrophages. And GALNT12-BMP signaling restained STAT3 signaling to regulate the proportion of M2 macrophage, DC, and NK cells to induce an immunosuppressive microenvironment in bone metastasis of PCa. However, the exact molecular mechanism through which GALNT12-BMP signaling modulated immune microenvironment to suppress bone metastasis of PCa warrants further exploration.

## Conclusion

Our results of this study illustrate the role and mechanism of GALNT12 in the process of bone metastasis of PCa. GALNT12 promotes the O-GalNAcylation of BMPR1A and then mediates the activation of BMP signaling. The activated BMP signaling could suppress the expression of integrin αVβ3 and the activation of STAT3 signaling. Integrin αVβ3 promotes the bone-specific metastasis of PCa via binding to vitronectin in bone matrix. Activated STAT3 signaling could modulate the immune microenvironment. These results identify GALNT12 as a potential therapeutic target for metastatic PCa.

## Supplementary Material

Supplementary methods, figures and tables.

## Abbreviations

PCa: prostate cancer
GALNT: polypeptide N-acetylgalactosaminyl transferases
OS: overall survival
AR: androgen receptor
PSA: prostate-specific antigen
GT1: Glycosyltransferase 1
VVA: Vicia villosa lectin
MNC: mononuclear cell
DC: dendritic cell
NK: natural killer
MDSC: myeloid-derived suppressor cell
CTC: circulating tumor cell
ECM: extracellular matrix
APC: antigen-presenting cell
TIME: tumor immune microenvironment
