# Supplementary material for: GALNT12 suppresses the bone-specific prostate cancer metastasis by activating BMP pathway via the O-glycosylation of BMPR1A
Source: Int J Biol Sci. 2024 Jan 27;20(4):1297–313. doi: 10.7150/ijbs.91925 (PMC10878148; doi:10.7150/ijbs.91925)
Supplement: Supplementary file 1 — Supplementary methods, figures and tables. [file ijbsv20p1297s1.pdf]

Supplementary Methods

Cell lines and cell culture

Mouse PCa cell line (RM1), human PCa cell line (DU145 and PC-3), mouse bone marrow stromal cell line (OP-9) and mouse macrophage cell line (RAW264.7) were purchased from the National Collection of Authenticated Cell Culture (Shanghai). STR profile was used to notarize the cell line (Guangzhou Cellcook Biotech, Guangzhou, China). RM1, DU145 and PC-3 cells were cultured in RPMI 1640 medium, OP-9 cells were cultured in α-MEM medium and RAW264.7 cells were cultured in DMEM medium. All the medium was added with 10% fetal bovine serum (FBS), 100 U/mL penicillin and 100 mg/mL streptomycin. Cells were cultured in 37 °C moist environment with 5% CO_2.

Plasmid, lentivirus and siRNA transfection

Avi-flag-tagged human GALNT12 plasmid was purchased from iGeneBio (Guangzhou, China) and myc-tagged human BMPR1A plasmid was kindly given by Dr. Bo Jiang. The human GALNT12 mutant plasmid, mouse GALNT12 and its mutant plasmid and BMPR1A mutant plasmid were constructed by YouBio (Hunan, China). Lipofectamine 2000 was used for plasmids transfection according to the instruction.

Luciferase lentivirus was purchased from Genechem (Shanghai, China) and shGALNT12-Luci lentivirus was purchased from Corues Biotechnology (Nanjing, China) (sequence of shRNAs was listed in **Table S1**). Human and mouse GALNT12 overexpressing lentivirus was produced via pLP1, pLP2 and pLP/VSVG lentivirus packaging system in 293T cells using lipofectamine 2000.

To construct stably expressed cells, target cells were infected with lentivirus for 12 h and selected with antibiotic for 4 days.

Small interfering RNA (siRNA) against human GALNT12 were purchased from Generay (Shanghai, China) and transfected into Lncap cells with INTERFERin$^{@}$ (Polyplus, France).

RNA sequencing and quantitative real-time PCR (qRT-PCR) analysis

Total RNA was extracted from RM1^{parental}, RM1^{LuM3} and RM1^{BM4c} cells during the

logarithmic growth phase. Firstly, mRNA was purified from total RNA using poly-T oligo-attached magnetic beads. Fragmentation was carried out using divalent cations under elevated temperature in an Illumina proprietary fragmentation buffer. First strand cDNA was synthesized using random oligonucleotides and Super Script II. Second strand cDNA synthesis was subsequently performed using DNA Polymerase I and RNase H. Remaining overhangs were converted into blunt ends via exonuclease/polymerase activities and the enzymes were removed. After adenylation of the 3′ ends of the DNA fragments, Illumina PE adapter oligonucleotides were ligated to prepare for hybridization. To select cDNA fragments of the preferred 400-500 bp in length, the library fragments were purified using the AMPure XP system (Beckman Coulter, Beverly, CA, USA). DNA fragments with ligated adaptor molecules on both ends were selectively enriched using Illumina PCR Primer Cocktail in a 15 cycle PCR reaction. Products were purified (AMPure XP system) and quantified using the Agilent high sensitivity DNA assay on a Bioanalyzer 2100 system (Agilent). The sequencing library was then sequenced on NovaSeq 6000 platform (Illumina) Shanghai Personal Biotechnology Cp. Ltd.

qRT-PCR was performed as described previously[1]. Briefly, the total RNA was extracted from cells using TRIzol reagent (Invitrogen, Waltham, USA) and reversely transcribed to cDNA using RT SuperMix (Vazyme, Nanjing, China). qRT-PCR was performed on QuantStudio™ 6 Flex System (Applied Biosystems, Foster City, CA) using SYBR Master Mix (Vazyme, Nanjing, China). The relative expression of mRNA was normalized to ACTB by the $2^{-\Delta Ct\Delta Ct}$ method and the primers purchased from Generay (Shanghai, China) were listed in **Table S1.**

Immunohistochemistry (IHC) and Hematein-eosin (H&E) staining

IHC and the evaluation of staining intensity were performed as described previously[1]. Briefly, slides with sections (3 μm) of paraformaldehyde fixed tissue specimens enrolled were stained according a standard procedure. The staining intensity was scored as 0 (negative), 1 (weak), 2 (intermediary) and 3 (strong), while the staining range was scored as 0 (0%), 1 (1–25%), 2 (26–50%), 3 (51–75%) and 4 (75–100%), which

combined and resulted in scores on a scale of 0–12. For CD206 staining, the positive cells were counted per field.

For H&E staining, slides were stained in hematoxylin for 1 min and eosin for1 min. All the slides were viewed by a light microscope (Leica DM1000, Leica Biosystems Inc., Heerbrugg, GER).

Western blot (WB) analysis

WB was performed as described previously[1]. Briefly, cells were lysed by RIPA buffer (Byeotime, Shanghai, China) which contains phosphatase inhibitor and protease inhibitor and the lysate was centrifuged at 12,000 g for 10 min. The supernatant was transferred into a new tube and boiled at 95 °C with loading buffer for 5 min. ACTB was selected as the reference protein. The information of antibodies used in this study was list in **Table S2.**

MTT assay

MTT assay was performed to evaluate the proliferation of PCa cells. 3,000 cells were seeded into 96-well plates. 10 μL MTT solution was added to the wells at different time points. After incubation for 2 h, the supernatant was removed. 100 μL DMSO was added to the wells to dissolve the purple formazan crystals subsequently and the plate was placed in a 37 °C environment for 30 min. Absorbance was measured at 490 nm, using a microplate reader (Tecan Infinite, M annedorf, Switzerland), absorbance 630 nm was selected as a reference.

Transwell migration and invasion assay

For migration assay, 1×10^5 cells in 200 μL FBS-free medium were added to the upper transwell chamber (8 μm, BIOFIL, Guangzhou, China) and incubated at 37 °C. After 24 h, the chamber was fixed with 4% paraformaldehyde (PFA) and stained with the crystal violet and the cells on the bottom of chamber were counted under a microscope. For invasion assay, 100 μL diluted matrigel (Corning, NY, USA) was spread on the bottom of upper transwell chamber and placed in 37 °C for 4 h and the following

procedures were the same as migration assay.

Wound healing assay

5×10^5 cells were seeded into a 6-well plate per well. When the density of cells reached about 90% confluence, an artificial wound was scratched with a 1 mL pipette tip. The floating cells were removed by gently washing with PBS for several times and FBS-free medium were added to the wells subsequently. Images were taken at 0h and appropriate end time to estimate migration abilities of cells.

Sphere-formation assay

3,000 cells were seeded into ultra-low attachment plates (Corning, NY, USA) and cultured in serum-free RPMI 1640 with 10 ng/mL epidermal growth factor (EGF) (Peprotech), 10 ng/mL fibroblast growth factor (FGF) (Peprotech), 1×B-27 (Gibco, ThermoFisher Scientific) and 1× N2 supeement (Gibco, ThermoFisher Scientific). Spheres more than 50 μm were calculated and photographed.

Clone formation assay

100 cells were inoculated into 12-well plate and cultured with complete medium for 10-14 days. Cells were then fixed with 4% PFA for 15 min and stained with the crystal violet for 10 min. The number of clones formed more than 50 cells was calculated

Cell cycle, apoptosis and anoikis assay

Cells during the logarithmic growth phase were collected and fixed with 75% ethanol in 4 °C overnight for cell cycle assay. Then cells were washed with PBS twice and then incubated with propidine iodide (PI) for 30 min and then tested by a flow cytometry (ACEA, Hangzhou, China).

For apoptosis assay, cells were cultured in FBS-free medium for 24h and then collected and washed with PBS twice. Cells were stained with Annexin V-FITC and PI (Vazyme, Nanjing, China) for 10 min. The apoptosis was tested by the flow cytometry.

For anoikis assay, 5×10^5 cells were seeded into ultra-low attachment plates and cultured with complete medium for 24h. The apoptosis of cells was tested by the flow

cytometry with the Apoptosis Kit (Vazyme, Nanjing, China).

Supplementary figure legend

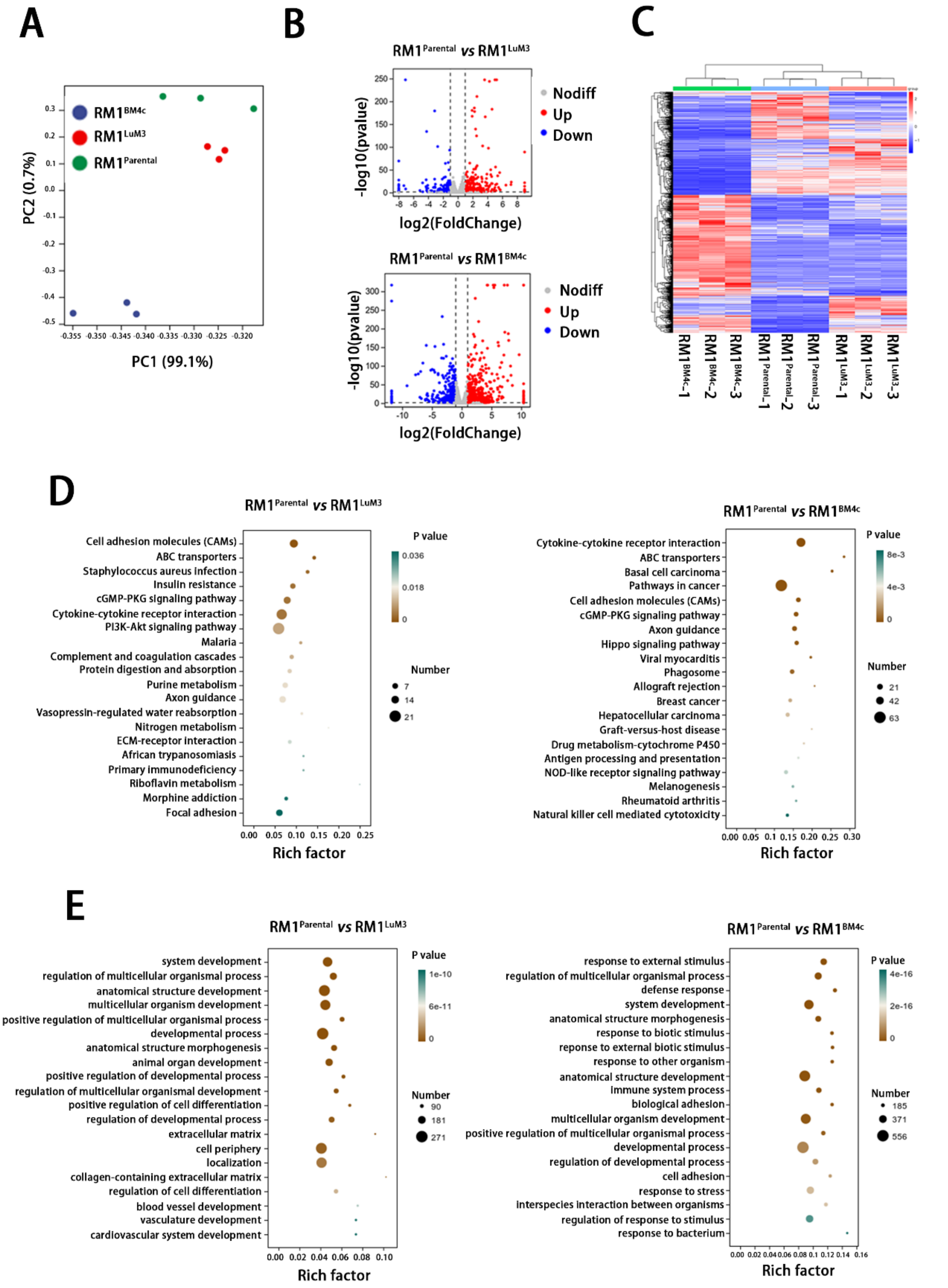

Figure S1. A: PCA analysis showing the profile of RM1^{parental}, RM1^{LuM3} and RM^{BM4c} cells. B: Volcano map analysis showing the differential expressing genes in RM1^{LuM3}

and RM^{BM4c} cells compared to $RM1^{parental}$ cells. C: Heat map analysis showing the genes expression between $RM1^{parental}$, $RM1^{LuM3}$ and RM^{BM4c} cells. D: KEGG enrichment analysis of differential expressing genes in $RM1^{LuM3}$ and RM^{BM4c} cells compared to $RM1^{parental}$ cells. E: GO enrichment analysis of differential expressing genes in $RM1^{LuM3}$ and RM^{BM4c} cells compared to $RM1^{parental}$ cells.

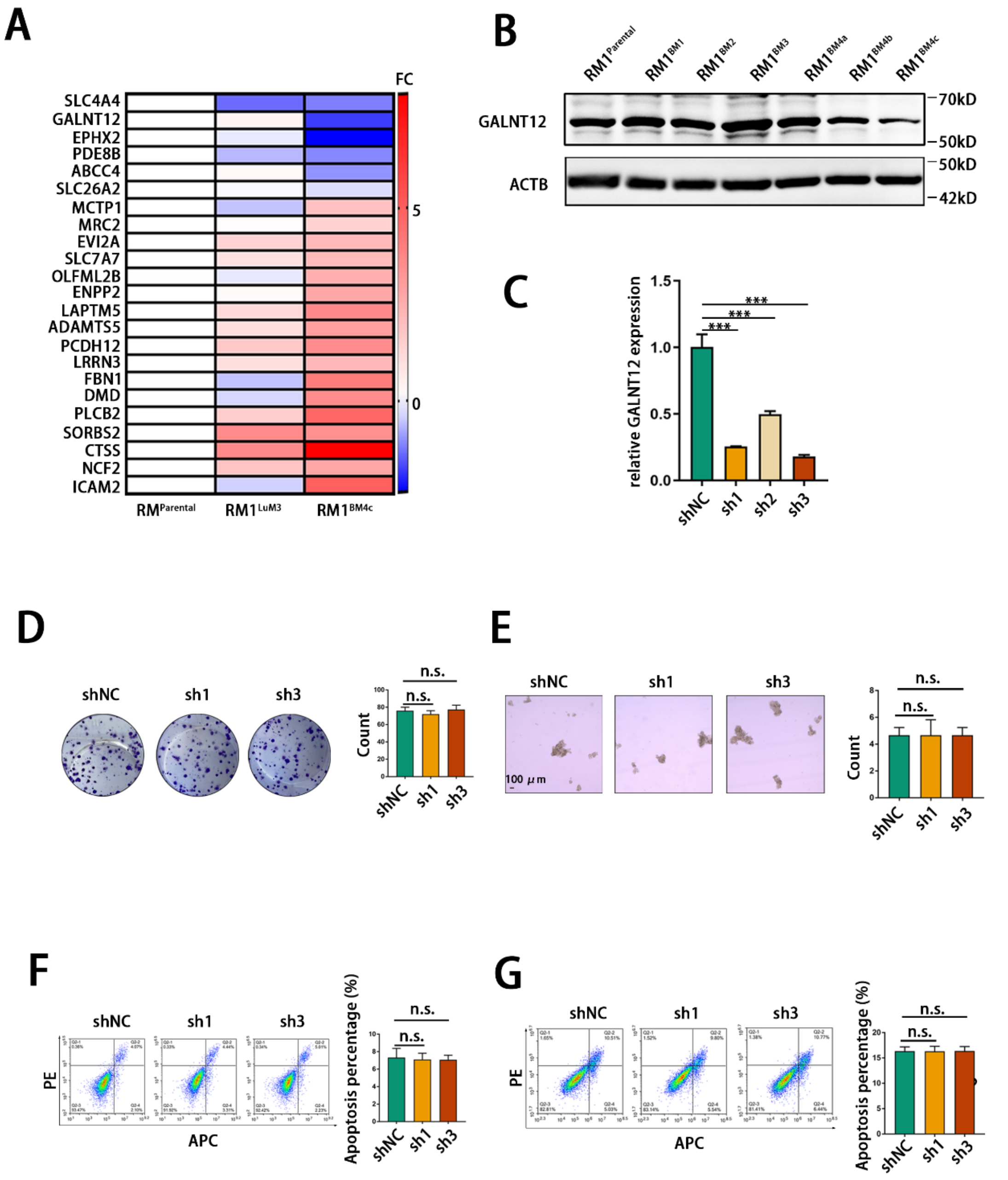

Figure S2. A: Heat map analysis showing expression of the selected 23 genes. B: The expression of GALNT12 in different bone metastatic derivatives was measured by WB. C: The knockdown efficiency of shRNA for GALNT12 in RM1 cells was verified by qRT-PCR. D: Representative clone formation assay images of control and GALNT12-

knockdown RM1 cells (left panel) and quantitation of the clone counts (right panel). E: Representative sphere formation assay images of control and GALNT12-knockdown RM1 cells (left panel) and quantitation of the sphere counts (right panel). F-G: Representative flow cytometry analysis images for apoptosis (F) and anoikis (G) (left panel) and quantitation of apoptotic cells (right panel).

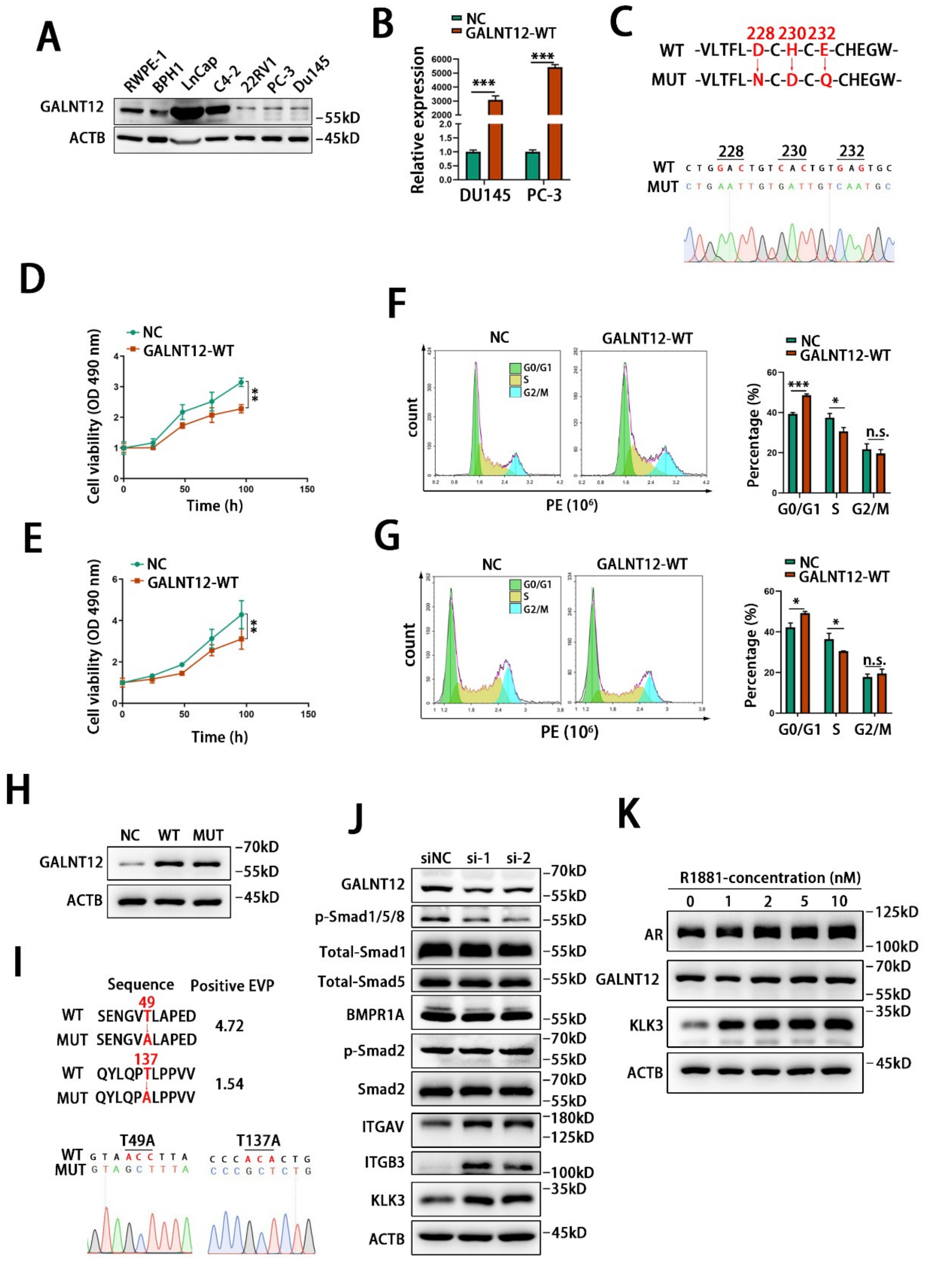

Figure S3. A: The expression of GALNT12 in human PCa cell lines. B: The efficiency of GALNT12 overexpressed WT vector was measured by qRT-PCR. C: The mutant sites of human GALNT12 (upper panel) and the verification by sanger sequencing (lower panel). D-E: MTT assay of control and GALNT12 overexpressed WT DU145

cells (D) and PC-3 cells (E) for proliferation. F-G: Flow cytometry assay of GALNT12 overexpressed WT DU145 (F) and PC-3 (G) cells for cell cycle (left panel) and quantitation of proportion of cell cycle (right panel). H: The efficiency of mouse GALNT12 overexpressed WT and MUT vector was measured by WB. I: The mutant sites of BMPR1A (upper panel) and the verification by sanger sequencing (lower panel). J: effect of GALNT12 expression on BMP and TGFβ signaling in AR-positive Lncap cells. K: effect of AR on GALNT12 expression in AR-positive Lncap cells.

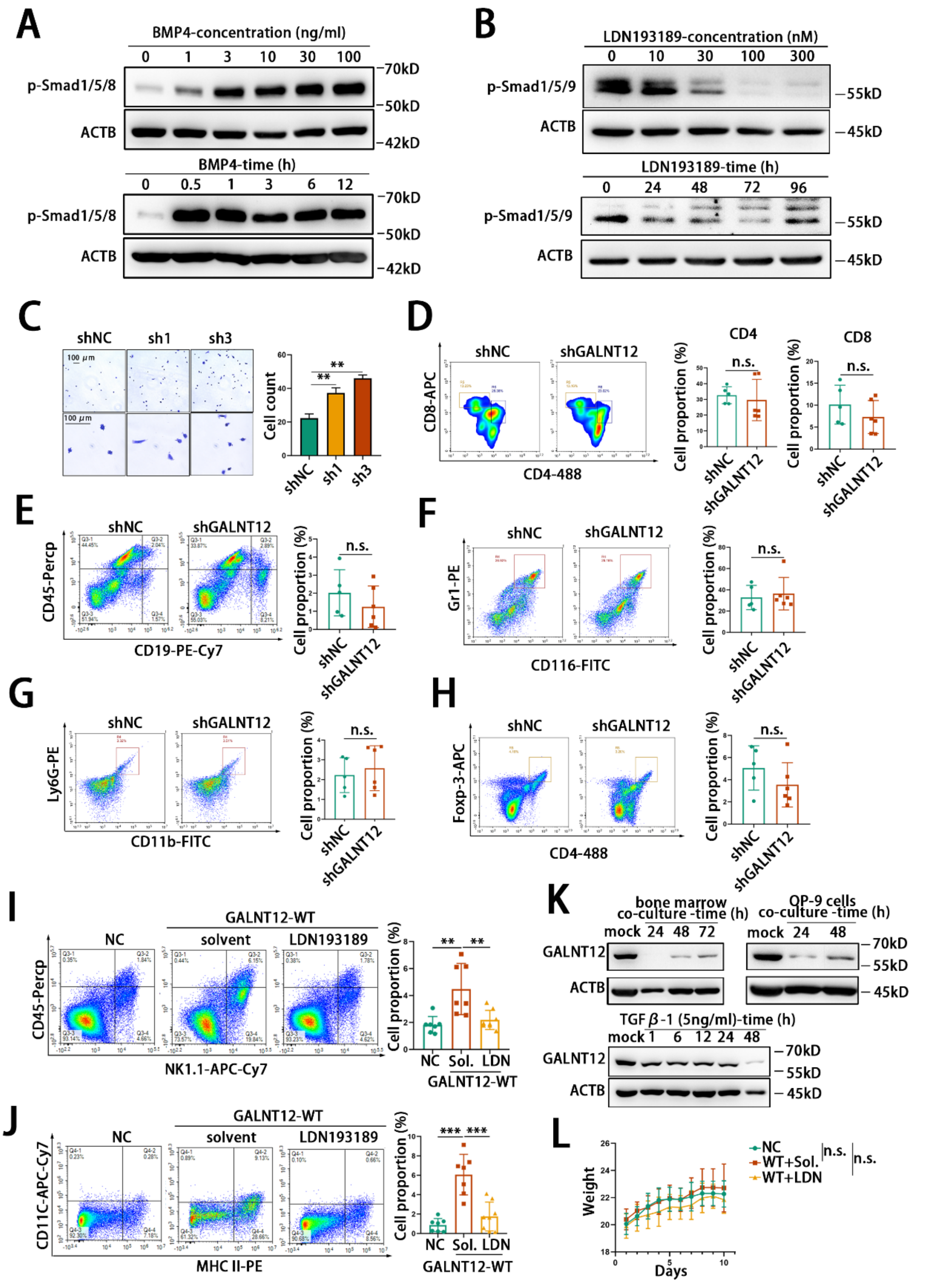

Figure S4. A: The level of p-Smad1/5/8 in BMP4 treating RM1 cells for indicated concentrations and times. B: The level of p-Smad1/5/9 in LDN193189 treating DU145 cells for indicated concentrations and times. C: Representative crystal violet staining images for the adhered ability of control and GALNT12-knockdown RM1 cells (left

panel) and quantitation of the adhered cells (right panel). D-H: Representative flow cytometry analysis images for proportion of T cells (D), B cells (E), MDSC cells (F), neutrophil (G) and Treg cells (H) in mouse bone metastatic lesion (left panel) and quantitation of cells proportion (right panel). I-J: Representative flow cytometry analysis images for proportion of NK cells (I) and DC cells (J) in GALNT12-WT overexpressed RM1^{BM4c} cells with or without LDN193189 treatment (left panel) and quantitation of cells proportion (right panel). K: The expression of GALNT12 in RM1 cell treated with bone marrow, OP-9 cells and TGFβ-1, respectively. L: The weight curve of mice with or without LDN193189 treatment.

Supplementary table

Table S1. Sequence of primers for qPCR and shRNA for GALNT12

Genes	Primer	Sequence
m-SLC4A4	Forward (5’-3’)	GAAGGTCACCACACGATCTACA
	Reverse (5’-3’)	TCCACATCAGATTTGTCGGAGT
m-GALNT12	Forward (5’-3’)	TCAACATCTATCTGAGCGACCG
	Reverse (5’-3’)	CTTGGGCAGGTTATCATAATCGT
m-EPHX2	Forward (5’-3’)	ACCACTCATGGATGAAAGCTACA
	Reverse (5’-3’)	TCAGGTAGATTGGCTCCACAG
m-PDE8B	Forward (5’-3’)	CAGGACCCTATCCAGGTTCTG
	Reverse (5’-3’)	GCGAGCGATGTTGCATCTG
m-ABCC4	Forward (5’-3’)	CATCGCGGTAACCGTCCTC
	Reverse (5’-3’)	CCGCAGTTTTACTCCGCAG
m-SLC26A2	Forward (5’-3’)	AAGAGCAGCATGACCTCTCAC
	Reverse (5’-3’)	CTGCCTCAAGTCAGTGCCT
m-MCTP1	Forward (5’-3’)	CCACAAGAACCTAAATCCTGTGT
	Reverse (5’-3’)	AAAGGCTGAGCCCATAAAGTC
m-MRC2	Forward (5’-3’)	ATCCAGGGAAACTCACACGGA
	Reverse (5’-3’)	GCGCTCATCTTTGCCGTAGT

m-EVI2A	Forward (5'-3')	ATGGAGCACAAAGGACAGTACC
	Reverse (5'-3')	CTAGCCCACAGGTGTGTGTA
m-SLC7A7	Forward (5'-3')	AGCACCAAGTATGAAGTGGCT
	Reverse (5'-3')	ACACGCCATTAAGCAGGGAG
m-OLFML2B	Forward (5'-3')	GTCTGGGGGTCCACTACAC
	Reverse (5'-3')	CTGGTTGTCCGCCTCGTTTT
m-ENPP2	Forward (5'-3')	ATGGCAAGACAAGGCTGTTTC
	Reverse (5'-3')	TTGACGCCGATGGCAAAAGT
m-LAPTM5	Forward (5'-3')	GATGCCGTACCTCAGGATGG
	Reverse (5'-3')	CTCCCGGTTCTTGACCACG
m-ADAMTS5	Forward (5'-3')	GGAGCGAGGCCATTTACAAC
	Reverse (5'-3')	CGTAGACAAGGTAGCCCACTTT
m-PCDH12	Forward (5'-3')	ACCATCCCAGACACAGAGGG
	Reverse (5'-3')	AGTCGCTTTACAGAAAGGTCCT
m-LRRN3	Forward (5'-3')	GATTGCCCCCAATTATGTACCTG
	Reverse (5'-3')	GGAGCAGAATCTGTGTGTCGG
m-FBN1	Forward (5'-3')	GGACGCCAATTTGGAGGCT
	Reverse (5'-3')	CTTTCAGCGCATCGTGTCCT
m-DMD	Forward (5'-3')	CGAGACCCAAACCACTTGTTG
	Reverse (5'-3')	GGTCAGCTAAAGACTGGTAGAGC
m-PLCB2	Forward (5'-3')	AGGATAGCTGTGATGGAAGAAGG
	Reverse (5'-3')	GCCCAGGTGTCAGGTATGTAG
m-SORBS2	Forward (5'-3')	ATGAATACAGATAGCGGTGGGT
	Reverse (5'-3')	TTTGGCCGAACGCTTCTAAAA
m-CTSS	Forward (5'-3')	CCATTGGGATCTCTGGAAGAAAA
	Reverse (5'-3')	TCATGCCCACTTGGTAGGTAT
m-NCF2	Forward (5'-3')	GCTGCGTGAACACTATCCTGG
	Reverse (5'-3')	AGGTCGTACTTCTCCATTCTGTA
m-ICAM2	Forward (5'-3')	TGGTCCGAGAAGCAGATAGTAG

	Reverse (5'-3')	GAGGCTGGTACACCCTGATG
m-ACTB	Forward (5'-3')	GGCTGTATTCCCCTCCATCG
	Reverse (5'-3')	CCAGTTGGTAACAATGCCATGT
h-GALNT12	Forward (5'-3')	GTGCGGCTGCACCAGATTA
	Reverse (5'-3')	AACTGTCCGAAGGAGAGTTGA
h-ACTB	Forward (5'-3')	CATGTACGTTGCTATCCAGGC
	Reverse (5'-3')	CTCCTTAATGTCACGCACGAT
m-ITGA4	Forward (5'-3')	AACCGGGCACTCCTACAAC
	Reverse (5'-3')	CACCACCGAGTAGCCAAACAG
m-ITGB1	Forward (5'-3')	ATGCCAAATCTTGCGGAGAAT
	Reverse (5'-3')	TTTGCTGCGATTGGTGACATT
m-ITGAV	Forward (5'-3')	CCGTGGACTTCTTCGAGCC
	Reverse (5'-3')	CTGTTGAATCAAACTCAATGGGC
m-ITGB3	Forward (5'-3')	CCACACGAGGCGTGAACTC
	Reverse (5'-3')	CTTCAGGTTACATCGGGGTGA
m-ITGA5	Forward (5'-3')	CTTCTCCGTGGAGTTTTACCG
	Reverse (5'-3')	GCTGTCAAATTGAATGGTGGTG
m-CXCR4	Forward (5'-3')	CTTCTGGGCAGTTGATGCCAT
	Reverse (5'-3')	CTGTTGGTGGCGTGGACAAT
Mouse-shGALNT12-1	Forward (5'-3')	gatccGCACCAGATCAACATCTATCTtcaagag AGATAGATGTTGATCTGGTGCtttttt
	Reverse (5'-3')	aattaaaaaaGCACCAGATCAACATCTATCT ctcttgaAGATAGATGTTGATCTGGTGCg
Mouse-shGALNT12-2	Forward (5'-3')	gatccGGACTGTACCAACTCAGATAAtcaagag TTATCTGAGTTGGTACAGTCCtttttt
	Reverse (5'-3')	aattaaaaaaGGACTGTACCAACTCAGATAA ctcttgaTTATCTGAGTTGGTACAGTCCg
Mouse-shGALNT12-3	Forward (5'-3')	gatccGCCCTGTTATCGATGTGATTGtcaagag CAATCACATCGATAACAGGGCtttttt

	Reverse (5'-3')	aattaaaaaGCCCTGTTATCGATGTGATTG<br>ctcttgaCAATCACATCGATAACAGGGCg
Human-siGALNT12-1	Forward (5'-3')	CAGUGUUCGUGCAGCUGAAGU
	Reverse (5'-3')	UUCAGCUGCACGAACACUGUU
Human-siGALNT12-2	Forward (5'-3')	GGGUAAUCGUAAGAUGUUAAC
	Reverse (5'-3')	UAACAUCUUACGAUUACCCUU

Table S2. Antibody

Antibodies	Source	Identifier
GALNT12	Biorbyt	Orb449042
BMPR1A	Invitrogen	38-6000
Anti-MYC	abcam	Ab9106
p-Smad1/5/9	CST	13820
Total-Smad1	CST	6944
Total-Smad5	Abclonal	A19117
p-Smad2	CST	18338
Total-Smad2	CST	5339
Anti-O-Link N-Acetylglucosamine	PTM Bio	PTM-952
ITGAV	Abclonal	A19071
ITGB3	Abclonal	A19073
STAT3	CST	9139
p-STAT3	CST	9145
β-actin	CST	3700
AR	Santa Cruz	sc-7305
KLK3	Proteintech	10679-1-AP
F4/80	abcam	5420
MHCII	Invitrogen	12-5321-81
CD206 (IHC/IF)	CST	24595
CD206 (Flow)	Invitrogen	53-2061-82
CD116	Invitrogen	11-1169-42

Gr-1	Biolegend	108408
CD45	Invitrogen	45-0451-82
NK1.1	BD Bioscience	560618
CD19	BD Bioscience	552854
CD11C	Biolegend	117324
CD4	Invitrogen	53-0041-82
CD8	Invitrogen	17-0081-82
CD11b	Invitrogen	53-0112-82
FOXP3	Invitrogen	17-5773-82

Table S3. The antibody for markers of different immune cells

Immune cell	Antibody for markers
Macrophage	APC-F4/80, PE-MHCII, Percp-CD206
MDSC	FITC-CD116, PE-Gr1, Percp-CD45
NK	APC-Cy7-NK1.1, Percp-CD45
B	PE-Cy7-CD19, Percp-CD45
DC	APC-Cy7-CD11C, Percp-CD45
T	PE-Cy7-CD3, 488-CD4, APC-CD8, Percp-CD45
Neutrophil	FITC-CD11b, PE-ly6G, Percp-CD45
Treg	488-CD4, APC-Foxp3, Percp-CD45

Reference

[1] Y. Yang, H. Qin, M. Ding, C. Ji, W. Chen, W. Diao, H. Yin, M. Chen, W. Gan, H. Guo, Small ankyrin 1 (sANK1) promotes docetaxel resistance in castration-resistant prostate cancer cells by enhancing oxidative phosphorylation, FEBS open bio, 13 (2023) 257-269.
